# An Intelligently Enhanced Ant Colony Optimization Algorithm for Global Path Planning of Mobile Robots in Engineering Applications

**DOI:** 10.3390/s25051326

**Published:** 2025-02-21

**Authors:** Peng Li, Lei Wei, Dongsu Wu

**Affiliations:** 1 College of Automobile and Traffic Engineering, Nanjing Forestry University, Nanjing 210037, China; 2 Department of Mechanical Engineering, National University of Singapore, Singapore 117575, Singapore; 3 College of Civil Aviation, Nanjing University of Aeronautics and Astronautics, Nanjing 210026, China

**Keywords:** mobile robots, ant colony optimization algorithm, global path planning, state transition probability

## Abstract

Global path planning remains a critical challenge in mobile robots, with ant colony optimization (ACO) being widely adopted for its swarm intelligence characteristics. To address the inherent limitations of ACO, this study proposes an intelligently enhanced ACO (IEACO) incorporating six innovative strategies. First, the early search efficiency is improved by implementing a non-uniform initial pheromone distribution. Second, the ε-greedy strategy is employed to adjust the state transition probability, thereby balancing exploration and exploitation. Third, adaptive dynamic adjustment of the exponents α and β is realized, dynamically balancing the pheromone and heuristic function. Fourth, a multi-objective heuristic function considering both target distance and turning angle is constructed to enhance the quality of node selection. Fifth, a dynamic global pheromone update strategy is designed to prevent the algorithm from prematurely converging to local optima. Finally, by introducing multi-objective performance indicators, the path planning problem is transformed into a multi-objective optimization problem, enabling more comprehensive path optimization. Systematic simulations and experimentation were performed to validate the effectiveness of IEACO. The simulation results confirm the efficacy of each improvement in IEACO and demonstrate its performance advantages over other algorithms. The experimental results further highlight the practical value of IEACO in solving global path planning problems for mobile robots.

## 1. Introduction

Mobile robots have become integral in various engineering applications due to rapid advances in technology. In complex environments, the ability to perform autonomous global path planning (that is, generating collision-free paths from a start point to a target while considering environmental constraints) is critical for ensuring effective navigation [1]. Global path planning not only underpins the intelligence of mobile robots but also plays a central role in ensuring safety and operational efficiency [2].

In industrial manufacturing, automated guided vehicles (AGVs) utilize global path planning to achieve efficient material handling and warehouse logistics, thereby optimizing production workflows and reducing operational costs. This technology has also revolutionized agricultural robotics, enabling autonomous agricultural vehicles to navigate complex field layouts while performing precision tasks such as seeding, spraying, and harvesting [3]. In the construction industry, mobile robots equipped with global path planning capabilities assist in site surveying, material transportation, and structural inspection tasks. These robots can safely navigate dynamic construction environments while avoiding both static obstacles and moving workers. In mining applications, autonomous vehicles similarly employ global path planning to navigate underground tunnels and open-pit environments, helping enhance operational safety and efficiency [4]. The healthcare sector has also benefited significantly from mobile robot path planning applications. Autonomous medical supply delivery robots in hospitals utilize global path planning to navigate corridor and avoid healthcare personnel and patients while ensuring timely delivery of critical supplies [5]. In disaster response scenarios, search and rescue robots employ path planning algorithms to navigate complex and unpredictable environments during search operations [6].

Traditional global path planning methods for mobile robots can be broadly categorized into classical and heuristic approaches. Classical methods, including Dijkstra’s algorithm, the A* algorithm, and artificial potential field (APF), have been widely implemented due to their mathematical foundations and guaranteed completeness [7]. However, these conventional methods often face significant limitations in complex engineering environments. While the Dijkstra and A* algorithms can effectively find optimal paths, their computational complexity grows exponentially with the scale of the environment. Similarly, APF methods frequently encounter problems with local minima and face difficulties in narrow passage navigation [8]. To address these diverse application requirements, researchers have increasingly turned to bionic algorithms, which draw inspiration from natural phenomena and biological systems. These methods, including genetic algorithms (GAs), particle swarm optimization (PSO), and ant colony optimization (ACO), have demonstrated exceptional capabilities in handling complex optimization problems [9]. Bionic algorithms offer advantages such as parallel computing capabilities, robust global search abilities, and strong adaptability to dynamic environments. Among these nature-inspired approaches, ACO has emerged as a particularly promising method for mobile robot path planning due to its inherent positive feedback, distributed computation, and heuristic search mechanisms [10]. The ACO algorithm’s ability to find optimal paths through indirect communication between simple agents (ants) makes it particularly suitable for navigation in complex engineering environments [11].

**Figure 1:**
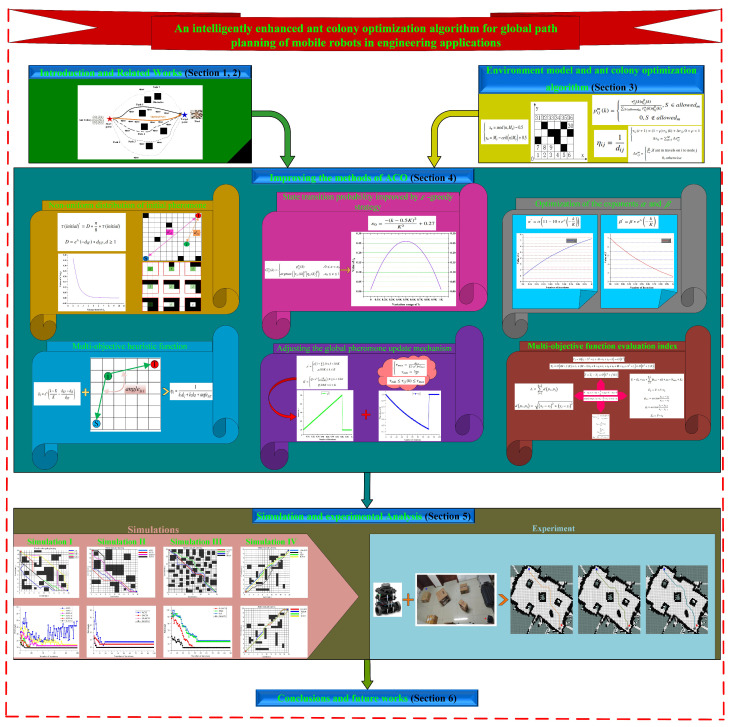
Article framework.

The remainder of this paper is organized as follows: Section 2 covers related works; in Section 3, the principles of grid maps and ACO are presented; our improvements to the ACO algorithm are introduced in Section 4; Section 5 describes the experiments and analyses; finally, conclusions and future research prospects are discussed in Section 6. The specific structure of the article is illustrated in Figure 1 below.

## 2. Related Works

In the field of engineering applications for mobile robots, global autonomous path planning is a crucial aspect in which algorithm research plays a significant role in improving path-planning methods [12]. Traditional path-planning algorithms such as the Dijkstra algorithm and rapid-exploration random tree (RRT) are commonly employed to achieve this objective [13]. A new model based on the Dijkstra algorithm was proposed by Resmiye Nasiboglu to generate public transport routes with the shortest path, minimum transfer criteria, and highest accessibility [14]. An enhancement technique known as PI-DP-RRT was subsequently proposed by Qiyong and colleagues; this technique incorporates automatic recognition system data and Douglas–Peucker compression to improve the sampling strategy of the algorithm through biased sampling. This work has led to shorter navigation paths for ships and overall improvements in safety [15]. Although the aforementioned algorithms have been improved, their inherent limitations are still challenging to address. For instance, Dijkstra’s algorithm guarantees the global shortest path but suffers from low efficiency, while RRT, though effective in high-dimensional environments, is limited by path quality and randomness.

Scientists have also developed various bioinspired algorithms for mobile robot path planning. These algorithms draw inspiration from biological and natural systems such as ant colonies, and include methods based on genetic algorithms and particle swarm optimization to solve complex path-planning problems [16]. One notable proposal was introduced by J. Zheng et al., who developed a distributed random algorithm that utilizes an enhanced genetic algorithm. Their approach overcomes the prevalent issue of other algorithms becoming stuck in local optima. This is achieved by implementing a point crossover operator and incorporating an anytime local search framework [17]. P.K. Das et al. utilized improved particle swarm optimization (IPSO) and improved the gravitational search algorithm (IGSA) to determine the optimal trajectory group for multiple robots in a cluttered environment. Their proposed approach combines the social nature of IPSO with the motion mechanism of IGSA. Their proposed hybrid IPSO-IGSA method maintains an effective balance between exploration and exploitation [18]. A number of hybrid algorithms have been widely studied as well. A hybrid meta-heuristic method was proposed in [19] to tune the parameters and aid in feature selection. In [20], a reliable and efficient GWO variant was proposed to improve the performance of CGWO via population diversity enhancement. A multilevel threshold image segmentation method using hybrid arithmetic optimization and the Harris Hawk optimization algorithm was proposed in [21]. Despite the improvements made to the algorithm, the inherent limitations of bioinspired and hybrid algorithms, such as a large number of parameters that complicates parameter tuning and high computational complexity, continue to hinder their widespread application in various scenarios.

**Figure 2:**
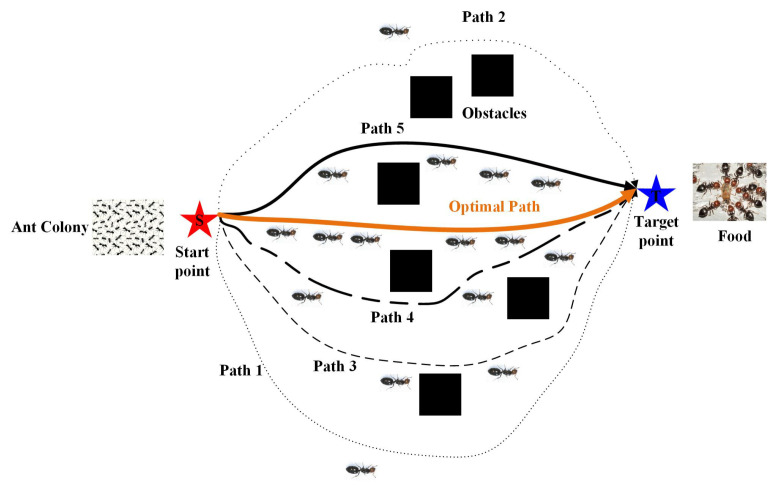
Schematic diagram of ant colony path search.

The ACO algorithm mimics the behavior of ants searching for food by releasing a chemical substance called pheromone, which is deposited along their travel path. Ants choose their next move based on the concentration of pheromone, which gradually dissipates through evaporation [22]. Figure 2 shows the schematic diagram of the ant colony path search. Researchers initially proposed the reinforced ACO in mobile robot path planning [23]. This algorithm involves solving a continuous function optimization problem with constraints, and offers a new solution to path planning problems. The ACO algorithm primarily focuses on transition probabilities and positive pheromone feedback for position improvements and optimizations [24]. Moreover, Yun Ni et al. noted the complexities of using a single algorithm to solve complex optimization problems. As a solution, they proposed a splitting strategy that incorporates local path or intelligent path optimization algorithms into global path planning to enhance the search efficiency and optimization quality [25]. Liu Zhonpu et al. proposed an improved ACO algorithm to address slow optimization speeds and redundant planning results in ACO-based mobile robot path planning. Their algorithm features fast optimization and composite prediction mechanisms, resulting in high precision [26].

Despite these advances, several challenges remain. Many existing ACO-based approaches still suffer from issues such as inefficient initial searches, lack of dynamic parameter adaptation, and suboptimal path refinement, especially when deployed in real-world scenarios. To address these limitations, this paper introduces the intelligently enhanced ant colony optimization algorithm (IEACO). The primary contributions of our work are as follows:

(1) By using a non-uniform distribution of initial pheromone, we adjust the initial pheromone distribution to more effectively guide the early search process.

(2) The state transition probability is improved by a ε-greedy strategy. By incorporating this ε-greedy strategy into the state transition probability, the proposed IEACO is better able to navigate the search space.

(3) By optimizing the α and β exponents, our proposed algorithm dynamically adjusts the exponents α and β to improve global search efficiency.

(4) We optimize the multi-objective heuristic function by integrating target information and turning angle considerations to more accurately guide the search.

(5) The global pheromone update mechanism is reformed in order to mitigate premature convergence.

(6) Finally, transforming the path planning problem into a multi-objective optimization framework allows the proposed IEACO to yield more precise and practical solutions.

## 3. Environment Model and Ant Colony Optimization Algorithm

### 3.1. Grid Map Mathematical Model

The construction of an environmental model is an important part of global path planning for mobile robots. Different methods are used to construct environment models in various engineering fields [27]. Commonly used methods include the octree method, grid method, topology method, and free space method. In this paper, considering the difficulty and practicality of different methods, the grid method is chosen to construct the environment [28]. The grid method has the advantages of simple structure and ease of implementation, which can significantly reduce the complexity of the environmental model. Moreover, the grid method is easy to maintain and update [29]. By using the grid method to construct an environment, a reliable foundation can be provided for the global path planning of mobile robots [30].

The map created with the grid method consists of two main parts. The white grids represent freely selectable grids, while the black grids represent obstacle grids. For example, consider a 6 × 6 square grid map. The grid cells are numbered from left to right and from top to bottom. When an obstacle is smaller than the grid, it is processed based on the occupied grid cell. The side length of a grid cell is the length of the robot’s movement step, which is the unit length.

**Figure 3:**
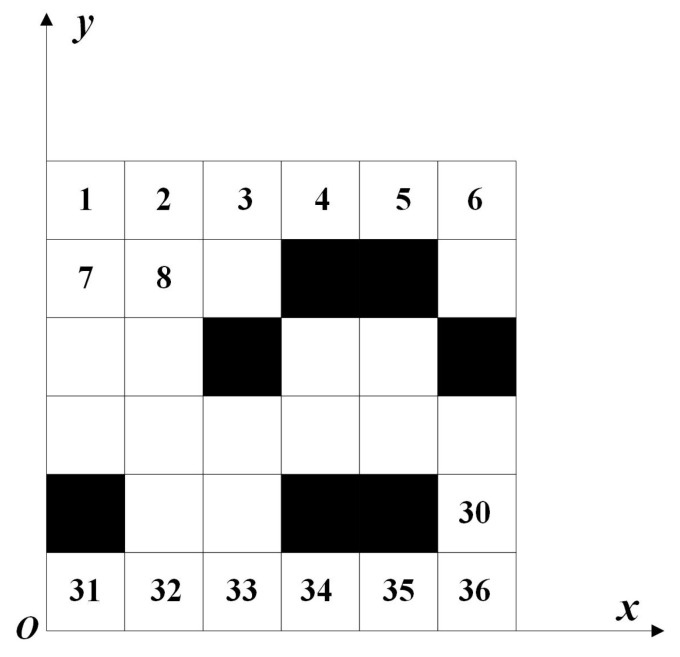
Grid map environmental model.

The distance between two grid cells is calculated based on the Euclidean distance between their midpoints, which is either the length of one cell or the length of $\sqrt{2}$ cells. For convenience, each cell is assigned a unique sequential number and coordinate value. The grid cells are numbered starting from the top left corner, and the conversion rules between the coordinates and the sequential grid cells numbers are shown in Figure 3.

The corresponding adjacency matrix records the identifier in each row, and the grid sequence number is converted to a coordinate $\left(x_{n},y_{n}\right)$ using Equation (1):

(1) $$\left\{\begin{array}{c} x_{n}=mod\left(n,M_{x}\right)-0.5\\ y_{n}=M_{y}-ceil\left(n/M_{y}\right)+0.5 \end{array}\right.$$

where n denotes the grid sequence number. The coordinates for the n-th grid are denoted as $\left(x_{n},y_{n}\right)$, where $x_{n}$ indicates the horizontal location and $y_{n}$ indicates the vertical location of the n-th grid cell in the environment. Here, $M_{x}$ represents the total number of rows in the environmental model, and $M_{y}$ represents the total number of columns. The functions ceil() and mod() refer to the round-down function and remainder function, respectively.

### 3.2. ACO Mathematical Model

The traditional ACO is a highly intelligent collective algorithm inspired by the foraging behavior of ants. In an ant colony, each ant is an independent yet cooperative individual, and their foraging behavior is executed through communication among individuals in the colony based on pheromone release. The ACO algorithm replicates the most effective behavior by simulating this process and using pheromone levels [31]. When ants search for food, they leave pheromone and create paths. Typically, pheromone evaporates over time, and the remaining pheromone concentration is greater on shorter paths than long paths [32]. Other ants tend to follow paths with higher pheromone concentrations based on their preferences. Over time, ants gather on the shortest route, creating a positive feedback effect [33]. ACO exhibits strong robustness and a good positive feedback mechanism in path planning, making it an ideal choice for determining the optimal path based on a global grid map.

Table 1 displays the configuration of the mathematical parameter model used to initialize the ACO algorithm. The fundamental parameters needed for ACO can be established by conducting experiments, and primarily consist of the total number of ants M released at each iteration k and the maximum number of iterations K.

The ACO algorithm searches for the optimal path starting from a certain grid node. The ants use the roulette wheel method to select an optional grid node from the many nodes they traverse until they reach the target grid node. The state transition probability is mainly based on the pheromone along each path and a heuristic function. The corresponding mathematical model is as follows (2):

(2) $$p_{ij}^{m}\left(k\right)=\left\{\begin{array}{c} \frac{\tau_{ij}^{\alpha }\left(k\right)\eta_{ij}^{\beta }\left(k\right)}{\sum_{S\in allowed_{m}}\tau_{ij}^{\alpha }\left(k\right)\eta_{ij}^{\beta }\left(k\right)},S\in allowed_{m}\\ 0,S\notin allowed_{m} \end{array}\right.$$

where k represents the k-th iteration and $\eta_{ij}$ is the heuristic function, which is expressed as shown in Equation (3):

(3) $$\eta_{ij}=\frac{1}{d_{ij}}$$

where $d_{ij}$ is the Euclidean distance from node i to node j.

As ants traverse a path, they leave behind a chemical trail with pheromone, which acts as a guide for fellow ants. The pheromone concentration is adjusted according to the path length, with the shortest paths having the highest pheromone levels. The ants communicate through pheromone, indirectly influencing the path choices. Through repeated iterations, ants eventually converge and locate the shortest path. Equations (4) and (5) provide the mathematical formula for pheromone updating, as follows:

(4) $$\left\{\begin{array}{c} \tau_{ij}\left(t+1\right)=\left(1-\rho \right)\tau_{ij}\left(k\right)+\Delta \tau_{ij},0<\rho <1\\ \Delta \tau_{ij}=\sum_{m=1}^{M}\Delta \tau_{ij}^{m} \end{array}\right.$$

(5) $$\Delta \tau_{ij}^{m}=\left\{\begin{array}{c} \frac{Q}{L_{m}},\text{if ant m travels from i to node j}\\ 0,otherwise \end{array}\right.$$

in which $\tau_{ij}\left(k+1\right)$ represents the (k + 1)-th pheromone, ρ is within (0, 1), $\left(1-\rho \right)\tau_{ij}\left(k\right)$ represents the pheromone left over from the k-th iteration, and $\Delta \tau_{ij}$ represents the pheromone increment.

## 4. Improving the Methods of ACO

To address the inherent limitations of the traditional ACO algorithm in terms of computational efficiency and convergence to local optima, in this section we propose the intelligently enhanced ant colony optimization algorithm (IEACO). The proposed algorithm improves the performance of ACO through the integration of six key advancements. First, an optimized initial pheromone distribution strategy is applied to enhance early-stage search efficiency. Second, an ε-greedy mechanism is incorporated into the state transition probability function to achieve a dynamic balance between exploration and exploitation. Third, adaptive methods for adjusting the heuristic function and pheromone intensity exponents are introduced, enhancing the algorithm’s adaptability across different scenarios. Fourth, a composite heuristic function based on target distance and turning angles is constructed to improve path evaluation accuracy. Fifth, an improved global pheromone update mechanism is designed to effectively reduce the risk of local optima entrapment. Finally, a multi-objective evaluation framework is established, transforming path planning into a multi-objective optimization problem that comprehensively considers factors such as path length, safety, energy consumption, and time complexity to achieve more thorough path optimization.

### 4.1. Non-Uniform Distribution of Initial Pheromone

**Figure 4:**
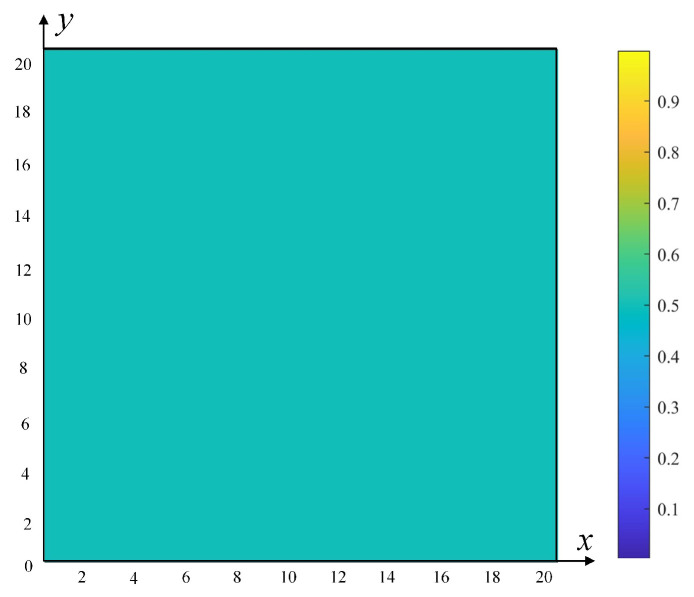
Uniform distribution of pheromone concentration.

**Figure 5:**
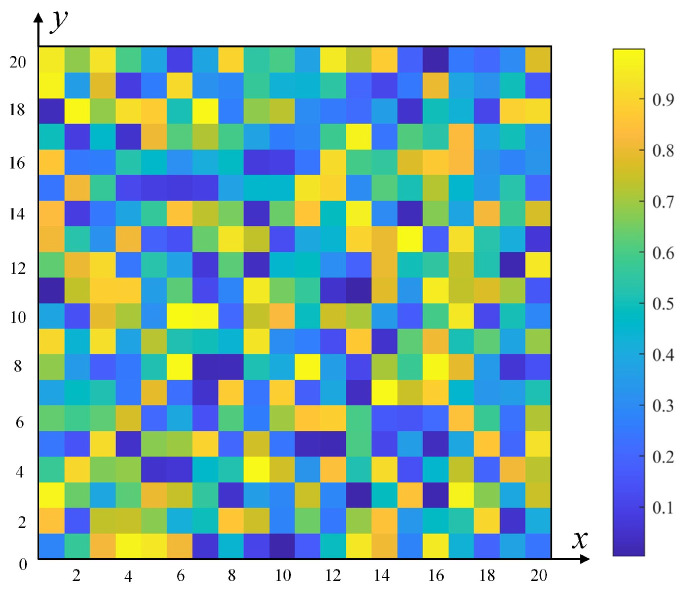
Non-uniform distribution of pheromone concentration.

**Figure 6:**
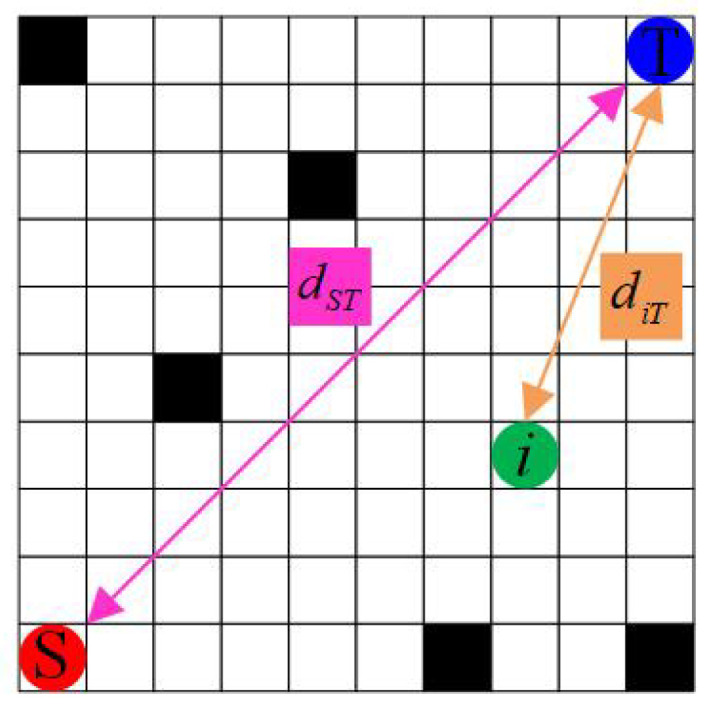
Calculation diagram of d.

The initial pheromone distribution plays a crucial role in the initial search path of ACO. As illustrated in Figure 4, traditional ACO employs a uniform distribution of initial pheromones in the grid map. This approach leads to numerous ineffective searches during the initial iterations, not only reducing convergence speed but also negatively impacting the algorithm’s overall efficiency and solution quality. To address this issue, this study proposes a non-uniform initial pheromone distribution strategy, depicted in Figure 5. This novel distribution strategy considers multiple key factors, including the distance from grid points to the target point, the distribution of surrounding obstacles, and the distance from the start point to the target point. The proposed non-uniform distribution of initial pheromone is mathematically formulated in Equation (6):

(6) $$\tau (initial{)}^{\prime }=D*\frac{n}{8}*\tau \left(initial\right)$$

(7) $$D=e^{\wedge }\left(-d_{iT}\right)*d_{ST},d\geq 1$$

where $\tau \left(initial\right)$ represents the initial pheromone concentration, $\tau (initial{)}^{\prime }$ denotes the improved pheromone concentration, D represents the distance information calculated using Equation (7), and n indicates the number of traversable free grid cells surrounding the current point *i*. In addition, $d_{iT}$ is the Euclidean distance from grid point *i* to the target point T, calculated as $d_{iT}=\sqrt{{\left(x_{i}-x_{T}\right)}^{2}+{\left(y_{i}-y_{T}\right)}^{2}}$, where the coordinates of *i* are $\left(x_{i},y_{i}\right)$, those of T are $\left(x_{T},y_{T}\right)$, and $d_{ST}$ represents the Euclidean distance from the start point S to the target point T, calculated as $d_{ST}=\sqrt{{\left(x_{S}-x_{T}\right)}^{2}+{\left(y_{S}-y_{T}\right)}^{2}}$, where the coordinates of S are $\left(x_{S},y_{S}\right)$. It is noteworthy that d≥1, where 1 represents the length of a single grid cell. Figure 6 shows an example calculation of d.

**Figure 7:**
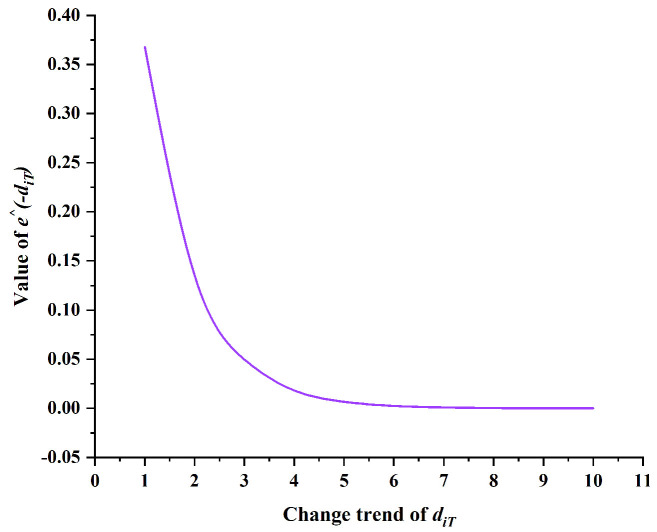
Change trend of $e^{\wedge }\left(-d_{iT}\right)$.

**Figure 8:**
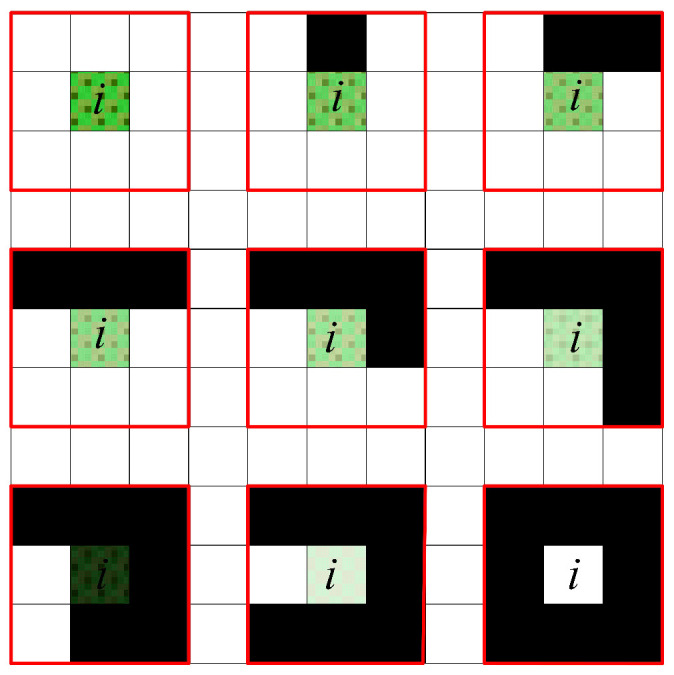
Distribution of obstacles around the grid.

This study proposes an innovative non-uniform pheromone initialization strategy that significantly enhances the ability of individuals to identify advantageous grids during ACO. Specifically, as illustrated in Figure 7, as the distance $d_{iT}$ from grid point *i* to target point T increases, the value of $e^{\wedge }\left(-d_{iT}\right)$ decreases, resulting in reduced pheromone concentration on grid *i* and consequently lowering its probability of selection. However, Figure 7 also reveals that as d increases, the rate of change in $e^{\wedge }\left(-d_{iT}\right)$ gradually plateaus. To address this issue and amplify the range of variation, we innovatively introduce the distance $d_{ST}$ as a coefficient in the calculation of D. Furthermore, this study considers the significant impact on path selection of obstacle distribution around grids. In Figure 8, black grids represent obstacles, while white grids indicate free spaces. Each grid is surrounded by eight adjacent grids, with the number of adjacent free grids positively correlating to the grid’s suitability as a path point. As the number of surrounding obstacles increases, the pheromone concentration on that grid gradually decreases, reflected by a reduction in grayscale value.

By integrating the distance information of the current grid and the distribution of surrounding grids, we optimize the initial pheromone into a non-uniform distribution. This method not only considers the traversability of grids but also reflects their potential advantages as path points. Therefore, this improvement increases the probability of the algorithm selecting advantageous areas in the initial stages, thereby enhancing its initial search efficiency.

### 4.2. Improved State Transition Probability Using the ε-Greedy Strategy

In traditional ACO, the roulette wheel selection mechanism is a crucial pathway for individual ants to move towards selectable points. However, the presence of numerous selectable nodes may significantly increase the computational complexity of traditional ACO. To address this issue, we introduce a deterministic state transition probability rule combined with an ε-greedy strategy to achieve a balance between exploration and exploitation. The improved state transition probability is presented in Equation (8):

(8) $$G_{ij}^{m}\left(k\right)=\left\{\begin{array}{cc} p_{ij}^{m}\left(k\right) & ,0\leq \varepsilon <\varepsilon_{0}\\ argmax\left\{{\left[\tau_{ij}\left(k\right)\right]}^{\alpha }{\left[\eta_{ij}\left(k\right)\right]}^{\beta }\right\} & ,\varepsilon_{0}\leq \varepsilon \leq 1 \end{array}\right.$$

where the argmax() function is used to determine the node j that maximizes the objective function f(j), where $f\left(j\right)={\left[\tau_{ij}\left(k\right)\right]}^{\alpha }{\left[\eta_{ij}\left(k\right)\right]}^{\beta }$, ε is an random variable distributed in the interval [0, 1] that controls the transition probability, and $\varepsilon_{0}$ is the threshold value of ε. When $\varepsilon_{0}\leq \varepsilon \leq 1$, ant m will move at time k+1 to the node that produces the maximum product of pheromone concentration and heuristic visibility. This deterministic selection mode facilitates faster convergence of the algorithm. Conversely, when $0\leq \varepsilon <\varepsilon_{0}$, ant m will select nodes according to the roulette wheel selection mechanism of the traditional ACO. The deterministic state transition probability rule explicitly defines the ant’s next action through a mathematical model, thereby reducing the computational burden associated with randomness. Concurrently, introduction of the ε-greedy strategy allows the algorithm to select the optimal path in most cases while retaining a certain probability of randomly choosing alternative paths. This strategy effectively balances the algorithm’s global search capability with its local optimization ability, enhancing overall algorithmic performance.

In fact, the value of $\varepsilon_{0}$ determines the probability of choosing between the deterministic selection mode and the random selection mode. Therefore, $\varepsilon_{0}$ significantly influences convergence speed and global search capability. If the value of $\varepsilon_{0}$ is small, the selection of the next point is more likely to be in deterministic mode. In this case, it can accelerate convergence speed; however, the global search capability will be reduced. If the value of $\varepsilon_{0}$ is large, then the path shift tends towards the random mode, which increases the randomness of path selection, resulting in increased computational complexity. Thus, it is necessary to propose rules for setting the value of $\varepsilon_{0}$ in order to balance the deterministic and random modes. Consequently, an adaptive adjustment mechanism for $\varepsilon_{0}$ is introduced to improve the state transition probability rule. The proposed formula for calculating $\varepsilon_{0}$ is shown in Equation (9).

(9) $$\varepsilon_{0}=\frac{-{\left(k-0.5K\right)}^{2}}{K^{2}}+0.27$$

**Figure 9:**
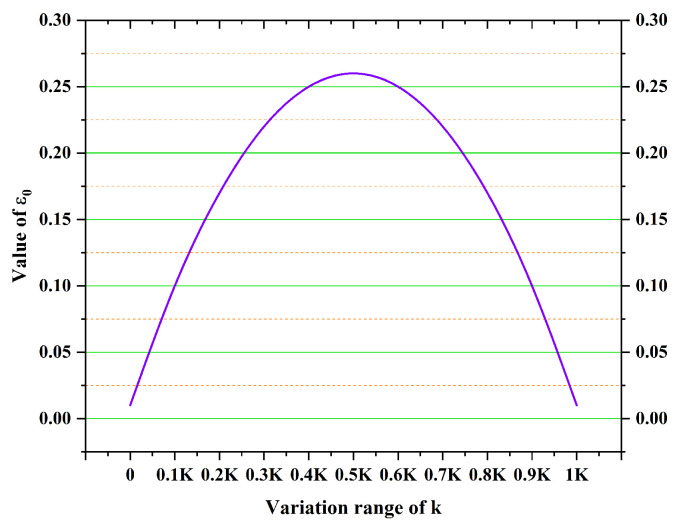
Adaptive change trend of $\varepsilon_{0}$.

In the early stages, $\varepsilon_{0}$ takes a smaller value, favoring deterministic transitions with higher probability, which can accelerate the search speed for locally optimal paths. During the algorithm’s execution, $\varepsilon_{0}$ takes larger values, increasing the probability of choosing random transitions, which avoids local optima. As the algorithm iterates to later stages and its evolutionary direction becomes essentially determined, the value of $\varepsilon_{0}$ can be gradually reduced to accelerate the convergence speed. Improving the state transition probability by using the ε-greedy method helps to achieve a good balance between global search capability and convergence speed. Figure 9 illustrates the adaptive adjustment for $\varepsilon_{0}$.

### 4.3. Optimization of the α and β Exponents

In the state transition probability of ACO, exponents α and β play crucial roles. These exponents respectively regulate the influence of the pheromone concentration $\tau_{ij}\left(k\right)$ and heuristic function $\eta_{ij}\left(k\right)$ on the path selection process of the ants. Traditional ACO methods typically employ fixed values for α and β (e.g., α=1, β=7) when addressing path planning problems [34]. However, this static parameter strategy may constrain the algorithm’s performance and adaptability. To enhance the efficiency and robustness of ACO, this study proposes a dynamic exponent adjustment method. We transform α and β from fixed constants into functions that evolve dynamically throughout the optimization process. This improvement enables the algorithm to adaptively adjust exponents based on the search state and iteration characteristics, thereby achieving a better balance between exploration and exploitation. The improved $\alpha^{\prime }$ and $\beta^{\prime }$ are shown in Equations (10) and (11).

(10) $$\alpha^{\prime }=\alpha \left(11-10*e^{\wedge }\left(-\frac{k}{K}\right)\right)$$

(11) $$\beta^{\prime }=\beta *e^{\wedge }\left(-\frac{k}{K}\right)$$

**Figure 10:**
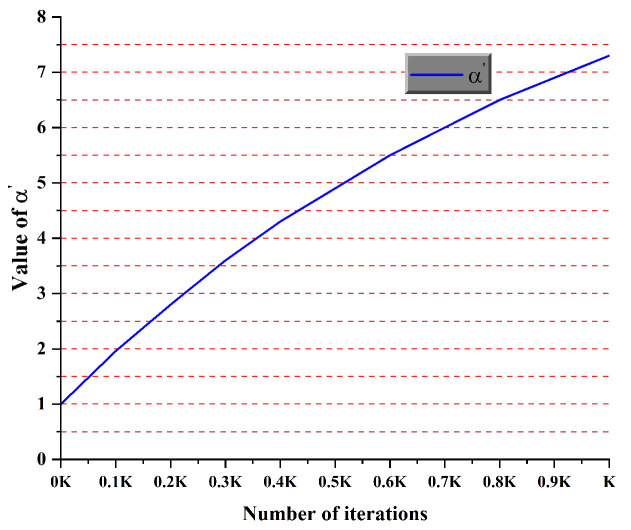
Change trend of $\alpha^{\prime }$.

**Figure 11:**
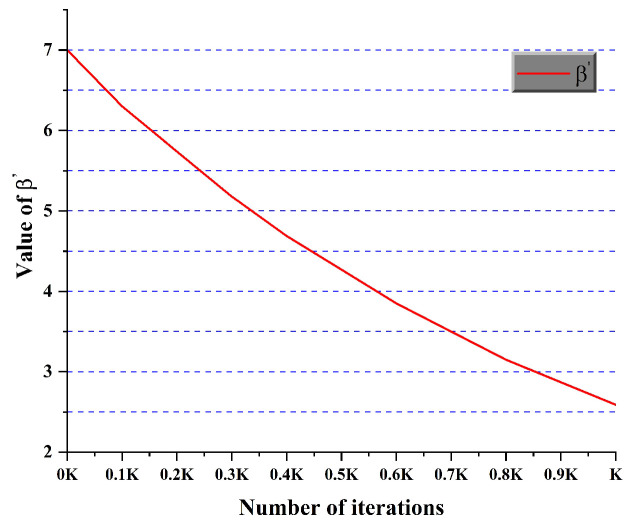
Change trend of $\beta^{\prime }$.

In this study, we implemented an adaptive balance between exploration and exploitation through dynamic adjustment of exponents α and β. Initially, a higher $\beta^{\prime }$ value and lower $\alpha^{\prime }$ value were employed, enhancing the heuristic function’s influence while reducing pheromone effects. This configuration promotes extensive solution space exploration by the ant colony, facilitating the discovery of potentially high-quality solutions and mitigating premature convergence to local optima. As the iterations progress, the parameter configuration gradually shifts, increasing $\alpha^{\prime }$ and decreasing $\beta^{\prime }$; this transition amplifies the pheromones’ guiding role, enabling more effective utilization of historical search experience and focusing exploitation on high-quality solution areas. Additionally, we adopted a dynamic adjustment mechanism based on an exponential function. This strategy ensures continuous and smooth parameter changes, enhancing the algorithm’s stability and robustness by effectively mitigating performance fluctuations that might arise from abrupt parameter alterations. Figure 10 and Figure 11 illustrate the change trends of $\alpha^{\prime }$ and $\beta^{\prime }$.

### 4.4. Multi-Objective Heuristic Function

**Figure 12:**
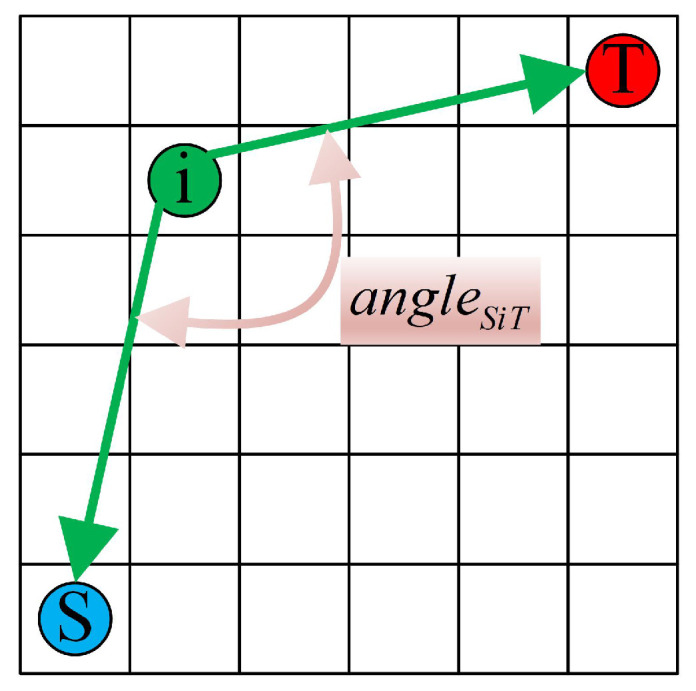
Calculation method of ${angle}_{SiT}$.

In traditional ACO, the heuristic function $\eta_{ij}\left(t\right)$ is typically calculated as the reciprocal of the Euclidean distance between the current node *i* and the next node *j*, denoted as $\frac{1}{d_{ij}}$; however, in a grid-based environment model, the distance between adjacent grids can be either 1 or $\sqrt{2}$. This causes the ants to be overly reliant on the heuristic function during the path search process, reducing the algorithm’s effectiveness and global search capability. To improve the algorithm’s global search efficiency, this study proposes an enhanced heuristic function; specifically, we incorporate the target point’s location information and the turning penalty factor into the heuristic function. The improved heuristic function formula is provided by Equation (12):

(12) $$\eta_{ij}=\frac{1}{\delta_{1}d_{ij}+\delta_{2}d_{iT}+{angle}_{SiT}}$$

where $\delta_{1}$ and $\delta_{2}$ are the respective weighting coefficients for $d_{ij}$ and $d_{iT}$, which are subject to the constraint $\delta_{1}+\delta_{2}=1$. The term ${angle}_{SiT}$ denotes the angle formed by the start point *S*, current point *i*, and target point *T*, as illustrated in Figure 12.

The improved heuristic function comprehensively considers both the target distance information and the turning angle. The smaller the distance and turning angle, the better the heuristic function. The dynamic adjustment strategy for $\delta_{2}$ (the weighting coefficient of $d_{iT}$) is shown in Equation (13). Given that the sum of $\delta_{1}$ and $\delta_{2}$ is always 1, uniquely determining one of them also determines the other. As iterations progress, the value of $\delta_{2}$ gradually increases, leading to an increase in the denominator of the heuristic function, thereby progressively reducing the heuristic function value. This mechanism effectively reduces unnecessary searches in the later stages of the algorithm, enhancing its computational efficiency. Based on this improved heuristic function, the algorithm can better balance local and global information when selecting the next node, helping to enhance the goal-oriented nature of the search. This improvement not only increases the search efficiency of the algorithm but also significantly enhances the smoothness of the planned path. In addition, ${angle}_{SiT}$ can further improve the path quality. Specifically, the ${angle}_{SiT}$ helps to penalize excessive turning, promoting smoother and more continuous paths. The inclusion of the ${angle}_{SiT}$ improves path smoothness by reducing sharp turns, which enhances the overall efficiency and stability of the planned trajectory. Moreover, it helps avoid unnecessary detours, leading to more direct paths.

(13) $$\delta_{2}=e^{\wedge }\left(\frac{k-K}{K}-\frac{d_{ST}-d_{iT}}{d_{ST}}\right)$$

### 4.5. Adjusting the Global Pheromone Update Mechanism

In global path planning problems, the pheromone update mechanism of the traditional ACO algorithm optimizes ant search behavior by dynamically adjusting path selection probabilities. This mechanism is primarily controlled by two key parameters: the evaporation coefficient ρ, and the pheromone intensity coefficient *Q*. The evaporation coefficient ρ suppresses the algorithm’s tendency to become trapped in local optima, while the intensity coefficient *Q* enhances the attractiveness of effective paths. However, the static nature of these two parameters in traditional ACO may limit the algorithm’s overall performance. As the iteration process progresses, excessive pheromone concentrations may accumulate on advantageous paths, causing more ants to favor these high-concentration paths. This phenomenon can significantly reduce the algorithm’s global search capability and increase the risk of becoming trapped in local optimal solutions. To overcome these limitations and enhance the algorithm’s robustness, this study proposes a method for dynamically adjusting the evaporation coefficient ρ and pheromone intensity *Q*. This method is implemented through Equations (14) and (15), aiming to maintain a balance between exploitation of known good solutions and exploration of the search space, which improves the algorithm’s ability to find global optimal solutions.

(14) $$\rho^{\prime }=\left\{\begin{array}{c} \rho \left(1+\frac{k}{K}\right),0\leq k<0.8K\\ \rho ,0.8K\leq k\leq K \end{array}\right.$$

(15) $$Q^{\prime }=\left\{\begin{array}{c} Q*e^{\wedge }\left(\frac{-4k}{K*ln\left(M\right)}\right),0\leq k<0.8K\\ Q,0.8K\leq k\leq K \end{array}\right.$$

**Figure 13:**
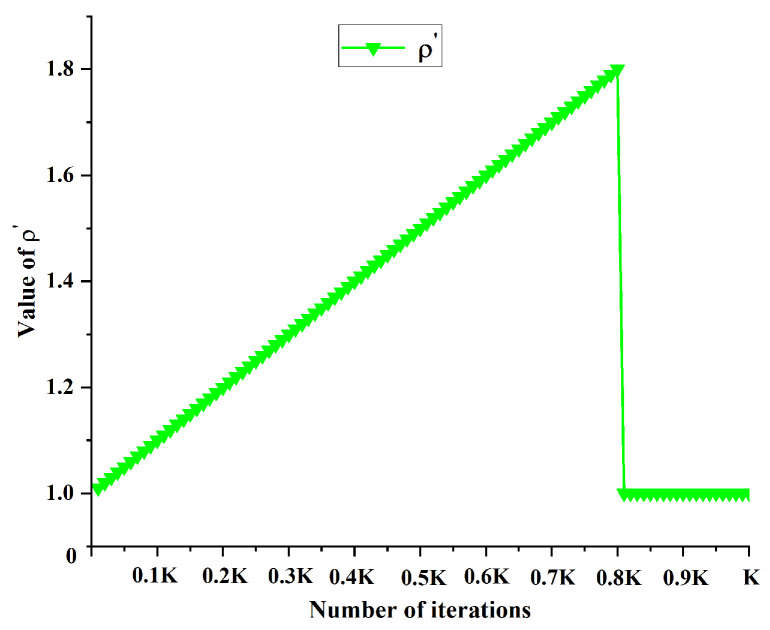
Range of variation of $\rho^{\prime }$.

**Figure 14:**
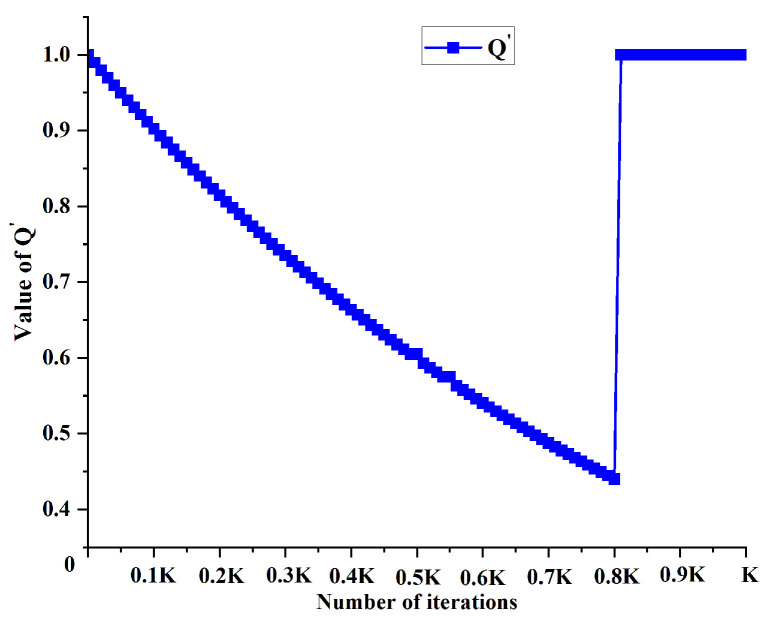
Range of variation of $Q^{\prime }$.

In the initial and intermediate stages of the algorithm, as the iteration count *k* increases, the improved evaporation coefficient $\rho^{\prime }$ exhibits an increasing trend while the improved intensity value $Q^{\prime }$ demonstrates a decreasing trend. This dynamic adjustment mechanism gradually reduces the pheromone levels during the path planning process, thereby encouraging the ant colony to explore new solution spaces and enhancing the algorithm’s global search capability. In the later stages of the algorithm, where the global optimal path has been successfully identified, the pheromone intensity and evaporation rate remain constant; this prompts the ant colony to favor the paths with higher pheromone concentrations, accelerating the algorithm’s convergence. Figure 13 and Figure 14 respectively illustrate the variation trends of the $\rho^{\prime }$ and $Q^{\prime }$ parameters.

In path planning applications utilizing ACO, the max–min ant system (MMAS) prevents the algorithm from converging to local optima by constraining the range of pheromone concentrations. Specifically, the MMAS imposes upper and lower bounds on the pheromone concentration $\tau_{ij}\left(k\right)$ for each edge, ensuring that the updated pheromone levels remain within the interval $\left[\tau_{min},\tau_{max}\right]$. This strategy mitigates excessive pheromone accumulation on certain paths, thereby enhancing the diversity of the search space and reducing the occurrence of local optima. This constraint is expressed in Equation (16):

(16) $$\tau_{min}\leq \tau_{ij}\left(k\right)\leq \tau_{max}$$

where $\tau_{min}$ and $\tau_{max}$ denote the minimum and maximum pheromone concentrations, respectively. Drawing from previous experimental findings, the calculation of $\tau_{max}$ and $\tau_{min}$ can be enhanced by incorporating iterative patterns and historical performance [35]. Equation (17) presents an improved formulation for computing $\tau_{max}$ and $\tau_{min}$:

(17) $$\left\{\begin{array}{c} \tau_{max}=\frac{d_{ST}}{\left(2-\rho^{\prime }\right)*L_{best}}\\ \tau_{min}=\frac{\tau_{max}}{N} \end{array}\right.$$

in which $L_{best}$ represents the minimal path length attained by an ant over *k* iterations. As demonstrated in Equation (17), incorporating the pheromone evaporation coefficient and optimal path length under the current iteration enhances the convergence efficiency of the ACO algorithm. This modification provides more effective guidance for node selection by individual ant colonies. Furthermore, integrationg $d_{ST}$ enhances the adaptability of $\tau_{max}$; in scenarios where $d_{ST}$ is substantial, indicating an expansive path search domain, the value of $\tau_{max}$ can be augmented to broaden the exploration space.

By adjusting the state transition probability and the global pheromone update mechanism, it is possible to get rid of the local minimum and increase effective exploration. Specifically, adaptive adjustment of the evaporation coefficient $\rho^{\prime }$ and pheromone intensity $Q^{\prime }$ encourages continual exploration during the early stages of the algorithm, preventing early stagnation. Additionally, resampling the adaptive variable ε at each iteration allows for a dynamic balance between deterministic (greedy) and probabilistic (explorative) selection, helping to avoid convergence to suboptimal solutions. Moreover, the transition criteria based on the current iteration *k*, with the threshold set at 0.8K (where *K* is the total number of iterations), have been experimentally validated; this adaptive threshold promotes a gradual shift from exploration to exploitation, enhancing the algorithm’s ability to escape local minima. The combination of these strategies significantly improves the algorithm’s robustness and ensures effective exploration, leading to higher-quality global solutions.

### 4.6. Multi-Objective Function Evaluation Index

In order to obtain a more accurate and effective solution, this study reformulates the path planning problem as a multi-objective optimization problem. We selected four key indicators as evaluation criteria: path length, safety, energy consumption, and time complexity. These indicators collectively constitute the constraints for multi-objective optimization of mobile robots, reflecting critical aspects such as path efficiency, reliability, energy efficiency, and computational performance [36]. Utilizing the TurtleBot3-Burger as the experimental platform, we conducted an in-depth analysis of these evaluation indicators to quantify the algorithm’s performance in practical application scenarios.

(1) Path Length Evaluation Index

Path length is a critical performance metric for mobile robots, as its value directly affects both the movement efficiency of the robot and the time required to complete tasks. The path length is shown in Equations (18) and (19):

(18) $$L=\sum_{i=1}^{n-1}d\left(p_{i},p_{i+1}\right)$$

(19) $$d\left(p_{i},p_{i+1}\right)=\sqrt{{\left(x_{i+1}-x_{i}\right)}^{2}+{\left(y_{i+1}-y_{i}\right)}^{2}}$$

where *L* represents the length of the path, *n* is the number of points on the path, $p_{i}\left(x_{i},y_{i}\right)$ and $p_{i+1}\left(x_{i+1},y_{i+1}\right)$ are adjacent points on the path, and $d\left(p_{i},p_{i+1}\right)$ denotes the distance between these adjacent points. In a grid map, if the movement between $p_{i}$ and $p_{i+1}$ is horizontal or vertical, then $d(p_{i},p_{i+1})=1$; if it is diagonal, then $d\left(p_{i},p_{i+1}\right)=\sqrt{2}$.

(2) Safety Evaluation Index

Safety can be reflected in the reliability of the path by quantifying the distance between the robot and obstacles. This metric aims to ensure that the robot remains as far away from obstacles as possible, achieving a collision-free path. Safety is directly related to the distance between the robot and the nearest obstacle. The calculation of the safety index is shown in Equations (20)–(23):

(20) $$D\left(p_{i}\right)=\left\{\begin{array}{cc} 1, & if\;p_{i}\;is\;dangerous\\ 0, & if\;p_{i}\;is\;safe \end{array}\right.$$

(21) $$D_{path}=\sum_{i=1}^{n}D\left(p_{i}\right)$$

(22) $$S=\frac{\sum_{i=1}^{n}D_{min}}{D_{path}+\varpi }$$

(23) $$D_{min}=\sqrt{{\left(x_{o}-x_{i}\right)}^{2}+{\left(y_{o}-y_{i}\right)}^{2}}$$

where $D_{\mathrm{path}}$ represents the number of hazardous grid cells along the path, *S* denotes the overall safety metric, and $D_{\min}$ is the minimum distance between each point on the path and the nearest obstacle. In addition, ϖ is a very small parameter to avoid $D_{path}$ being 0, while $\left(x_{o},y_{o}\right)$ is the coordinate value of the obstacle closest to $p_{i}$.

(3) Energy Consumption Evaluation Index

Energy consumption evaluation plays a crucial role in path planning, directly impacting the endurance of mobile robots. This study considers the path length, path angular variation, and turning frequency as the primary influencing factors, while also accounting for the energy consumption required for rotation during the motion of the TurtleBot3-Burger robot. The comprehensive energy consumption indicator can be represented by Equations (24)–(28):

(24) $$E=E_{L}+\mu_{1}*\sum_{i=2}^{n-1}\left|\phi_{i+1}-\phi_{i}\right|+\mu_{2}*N_{turn}+E_{\theta }$$

(25) $$E_{L}=U*I*t_{L}$$

(26) $$\phi_{i+1}=arctan\frac{y_{i+1}-y_{i}}{x_{i+1}-x_{i}}$$

(27) $$\phi_{i}=arctan\frac{y_{i}-y_{i-1}}{x_{i}-x_{i-1}}$$

(28) $$E_{\theta }=P*t_{\theta }$$

in which *E* represents the total energy consumption, where $E_{L}$ is the energy consumed due to the path length, *U* is the voltage, *I* is the current, and $t_{L}$ is the motion time with $t_{L}=L/v$, where *v* is the linear velocity. In addition, $\sum_{i=2}^{n-1}\left|\phi_{i+1}-\phi_{i}\right|$ is defined as the angle change between adjacent path segments $\phi_{i+1}$ and $\phi_{i}$, $N_{\mathrm{turn}}$ represents the total number of turns along the path, $\mu_{1}$ and $\mu_{2}$ are the respective weight coefficients of the path angle change and number of turns, $E_{\theta }$ represents the rotational energy consumption, *P* is the rotational power, and $t_{\theta }$ is the rotation time, calculated as $t_{\theta }=\theta /w$, where θ is the rotation angle and ω is the angular velocity.

(4) Time Complexity Evaluation Index

In global path planning for mobile robots based on ACO, the algorithm’s time complexity is primarily determined by the path generation time, which depends on both the initialization of the state matrix parameters and the path exploration process [37].

The state matrix initialization time can be expressed as follows:

(29) $$T_{1}=O\left(t_{0}+S^{2}*t_{1}+M*t_{2}+t_{3}*S\right)=O\left(S^{2}\right)$$

where *S* is the dimensionality of the space and *M* is the number of ants. The assumed initialization time for the algorithm’s information parameters is denoted as $t_{0}$, while the time required to initialize the pheromone matrix is $S^{2}*t_{1}$, the initialization time for each ant is $M*t_{2}$, and the time to initialize the path states is $t_{3}*S$.

The time of the path exploration phase can be expressed as follows:

(30) $$T_{2}=O\left[\left(M*f\left(S\right)\right)+t_{4}+\left(M-1\right)t_{5}+8*t_{6}+t_{7}+t_{8}+t_{9}+M*t_{10}+S^{2}*t_{1}\right]=O\left[S^{2}+f\left(S\right)\right]$$

where *K* is the maximum number of iterations, M∗f(S) is the time to calculate the fitness function, $t_{4}$ is the time to select the optimal individual, $\left(M-1\right)*t_{5}$ is the time to replace the final individual in the iteration, $8*t_{6}$ is the time to compute the next potential grid point on the path, $t_{7}$ is the computation time for the improved state transition function, $t_{8}$ is the dynamic adjustment time for pheromone evaporation rate and intensity, and $t_{9}$ is the time to generate random numbers. The time required to select the next node position is $M*t_{10}$, and the time to update the pheromone is $S^{2}*t_{1}$.

In summary, the time *T* of the algorithm can be expressed as

(31) $$T=T_{1}+T_{2}=O\left[S^{2}+f\left(S\right)\right].$$

In the application of path planning for the TurtleBot3-Burger robot, multiple performance indicators exhibit complex interactions and potential conflicts, forming a multidimensional optimization problem. Specifically, four key indicators are mutually constraining, namely, the path length, energy consumption, safety, and time complexity, constituting a highly coupled system. For instance, while the shortest path may minimize time, it could lead to increased energy consumption and reduced safety; conversely, overemphasizing safety might extend path length, thereby increasing both energy consumption and time. Consequently, the path optimization process requires a method capable of dynamically balancing these competing objectives. To address this multi-objective optimization problem, this study proposes a weighted comprehensive evaluation method. The proposed method can be represented by the following formula:

(32) $$R=h_{L}*L+h_{S}*\frac{1}{S}+h_{E}*E+h_{T}*T$$

where $h_{L}$, $h_{S}$, $h_{E}$, and $h_{T}$ represent the weight coefficients for the four key performance indicators. This weighted combination method provides a flexible framework capable of effectively handling the complex interrelationships and potential conflicts among various indicators. The determination of weight coefficients is a crucial parameter tuning process, typically based on the specific characteristics of the robot’s working environment, the particular requirements of the task, and the associated constraints.

By adjusting the weight coefficients, this method can flexibly balance different performance indicators according to the requirements of specific application scenarios, including the path length, energy consumption, and safety. This customizability allows for precise tuning of the weights based on the characteristics of the TurtleBot3-Burger and its operational environment, thereby adapting to diverse task requirements. This multi-objective optimization approach provides a more comprehensive and flexible solution for robot path planning in complex environments, with the potential to significantly enhance the overall performance of the TurtleBot3-Burger across various application scenarios. Therefore, the robot’s path planning problem can be formalized as a constrained multi-objective optimization problem, expressed as follows:

(33) $$minR=min(h_{L}*L+h_{S}*\frac{1}{S}+h_{E}*E+h_{T}*T),$$

**Figure 15:**
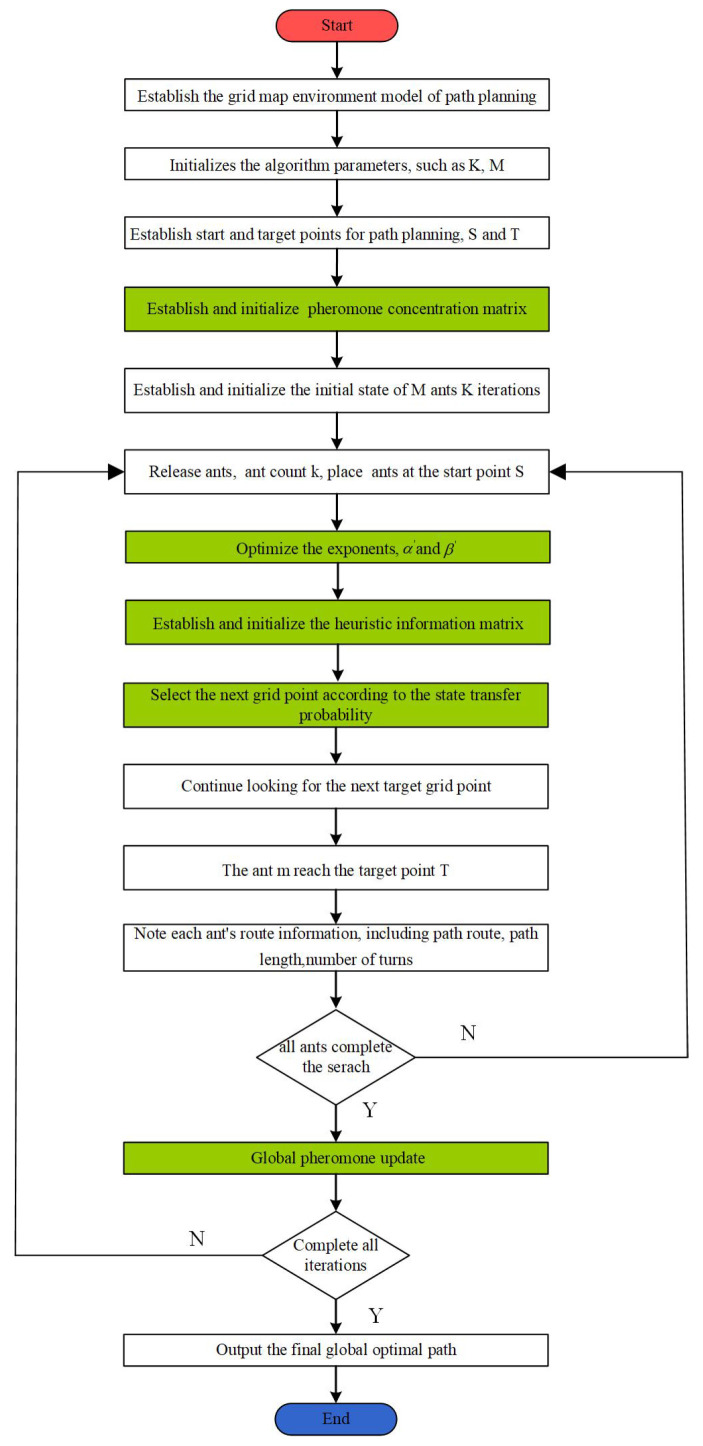
Flowchart of IEACO.

Algorithm 1 provides the pseudocode for IEACO, while the IEACO flowchart is shown in Figure 15.
**Algorithm 1** Process based on IEACO**Input:** initialize the parameters, such as K, M, α, β, Q, and ρ, as well as the heuristic information matrix, path matrix, and pheromone concentration;1:Build a grid map environment model;2:Initialize the parameters and build the start grid S and the target grid T;3:${\tau \left(initial\right)}^{\prime }=D*\frac{n}{8}*\tau \left(initial\right)$; $D=e^{\wedge }\left(-d_{iT}\right)*d_{ST},d\geq 1$;4:Arrange all ants at the designated start point S to initiate the path planning process;5:**for** k = 1 to K **do**6:  **for** m = 1 to M **do**7:   **while** ant m does not arrive at the target grid T **do**8:    Optimize the exponents $\alpha^{\prime }$ and $\beta^{\prime }$;9:    $\alpha^{\prime }=\alpha \left(11-10*e^{\wedge }\left(-\frac{k}{K}\right)\right)$, $\beta^{\prime }=\beta *e^{\wedge }\left(-\frac{k}{K}\right)$;10:    Implement the heuristic mechanism with target information and angle;11:    $\eta_{ij}=\frac{1}{\delta_{1}d_{ij}+\delta_{2}d_{iT}+{angle}_{SiT}}$;12:    Select the next optional grid by state transition probability;13:    $G_{ij}^{m}\left(k\right)=\left\{\begin{array}{cc} p_{ij}^{m}\left(k\right) & ,0\leq \varepsilon <\varepsilon_{0}\\ argmax\left\{{\left[\tau_{ij}\left(k\right)\right]}^{\alpha^{\prime }}{\left[\eta_{ij}\left(k\right)\right]}^{\beta^{\prime }}\right\} & ,\varepsilon_{0}\leq \varepsilon \leq 1 \end{array}\right.$14:   **end while**15:   **if** All the ants have successfully reached the target grid T **then**16:    Update global pheromone on all effective paths;17:    $\rho^{\prime }=\left\{\begin{array}{c} \rho \left(1+\frac{k}{K}\right),0\leq k<0.8K\\ \rho ,0.8K\leq k\leq K \end{array}\right.$, $Q^{\prime }=\left\{\begin{array}{c} Q*e^{\wedge }\left(\frac{-4k}{K*ln\left(M\right)}\right),0\leq k<0.8K\\ Q,0.8K\leq k\leq K \end{array}\right.$, $\tau_{min}\leq \tau_{ij}\left(k\right)\leq \tau_{max}$, $\left\{\begin{array}{c} \tau_{max}=\frac{d_{ST}}{\left(2-\rho^{\prime }\right)*L_{best}}\\ \tau_{min}=\frac{\tau_{max}}{N} \end{array}\right.$18:   **end if**19:  **end for**20:**end for****Output:** The final optimal path;

In general, compared with traditional ACO, the innovations of this paper are as follows: (1) a non-uniform initial pheromone distribution to enhance search efficiency; (2) an adaptive ε-greedy state transition mechanism that effectively balances exploration and exploitation; (3) dynamic adjustment of the α and β exponents to improve adaptability to varying conditions; (4) a multi-objective heuristic function that considers both distance and turning penalties, guiding the search towards smoother paths; (5) an adaptive global pheromone update mechanism that ensures continued exploration while focusing on promising paths; and (6) transformation into a multi-objective optimization problem, optimizing not only path length but also safety, energy consumption, and computational efficiency.

## 5. Simulation and Experimental Analysis

In this section, the performance advantages of IEACO in global path planning of mobile robots are validated through systematic simulations and experiment. Four simulations are employed to validate the effectiveness of each improvement integrated into the proposed approach, the robustness of IEACO under varying complexity levels on 20×20 grid maps, the algorithm’s performance on larger 30×30 grid maps, and its performance advantages compared to other well-known methods. In addition, the experimental evaluation is implemented on the robot operating system (ROS) platform using a TurtleBot3-Burger mobile robot, and comparative studies with commonly used path planning algorithms in practical engineering applications are conducted to validate the performance advantages of IEACO in real-world scenarios.

### 5.1. Simulation I

**Figure 16:**
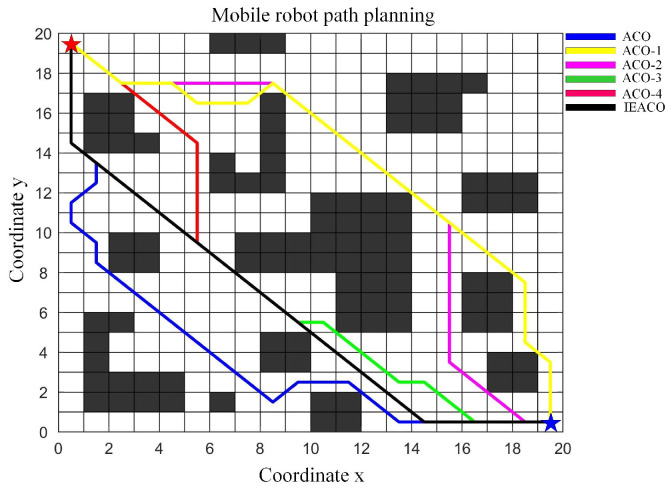
Optimal paths obtained by the six algorithms.

To validate the effectiveness of the proposed improvement methods, this study progressively combined these methods with traditional ACO in order to construct a series of algorithm variants, sequentially named ACO-1, ACO-2, ACO-3, ACO-4, and IEACO (as shown in Table 2). The simulation was conducted in a 20×20 grid map testing environment containing multiple obstacle traps, as illustrated in Figure 16. In the figure, the start point S and target point T are marked with red and blue pentagrams with grid coordinates at (0.5,19.5) and (19.5,0.5), respectively, corresponding to grid indices 1 and 400. The initial parameter settings for each algorithm variant are presented in Table 3.

**Figure 17:**
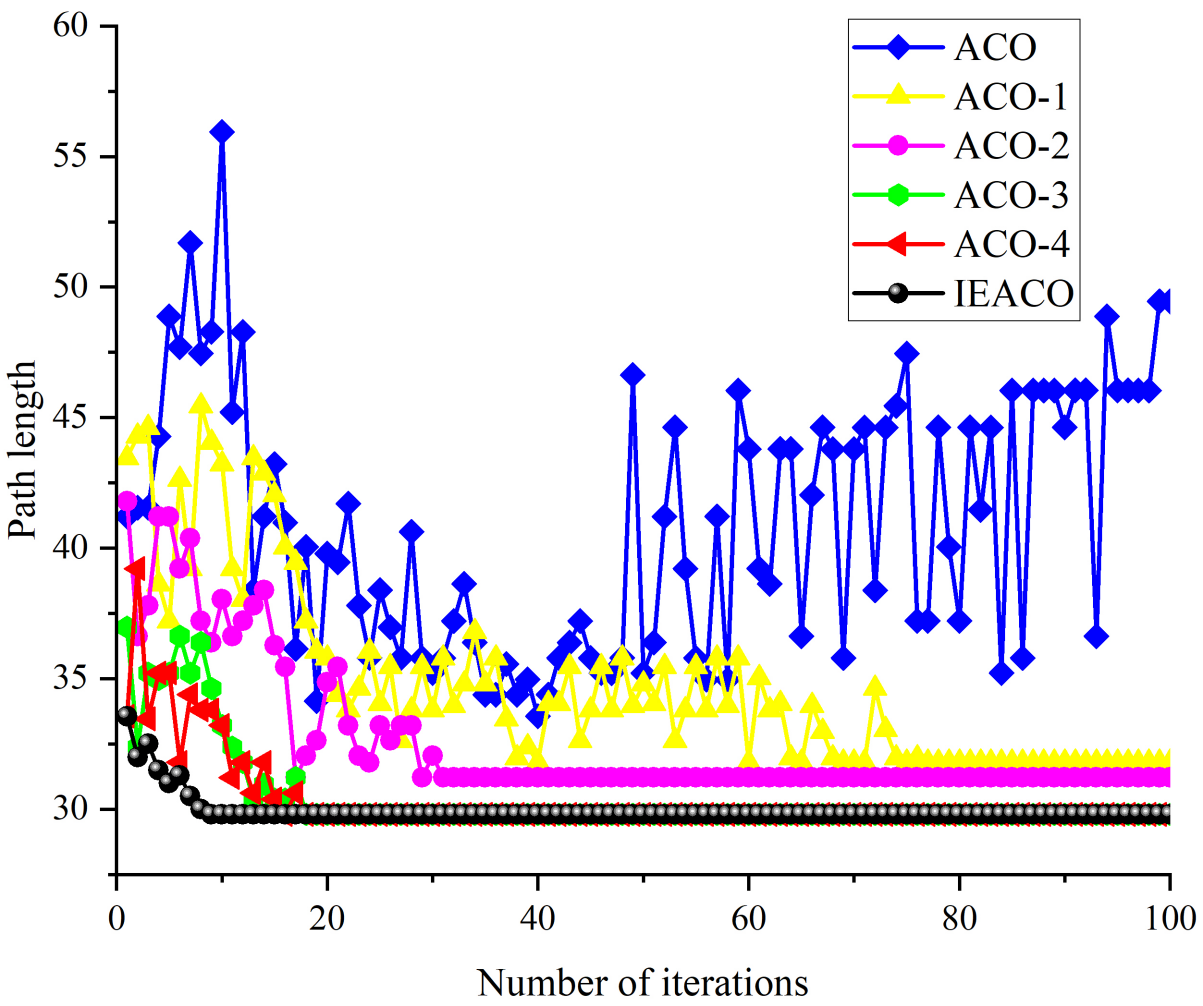
Convergence iteration curves of the six algorithms.

Considering the stochastic characteristics of intelligent optimization algorithms, solutions obtained from single searches may exhibit fluctuations; therefore, this study employs statistical metrics for performance evaluation, with each algorithm independently executed ten times to solve an identical path planning problem. The optimal paths obtained by each algorithm and their convergence curves are shown in Figure 16 and Figure 17, respectively, with detailed performance comparison results summarized in Table 4.

The simulation results demonstrate the progressive performance improvement of the ACO through sequential enhancements in path planning. Initially, the introduction of non-uniform initial pheromone distribution (ACO-1) reduces the optimal path length from 33.556 to 31.798, decreases the average path length from 34.675 to 33.986, and improves path smoothness by reducing the number of turns from 11 to 8.

Further enhancement of the state transition probability (ACO-2) continues to improve algorithm performance, reducing the optimal path length to 31.213, lowering the average path length to 33.142, and significantly improving convergence speed (from 72 to 31) while further reducing the number of turns to 5. Subsequently, optimization of pheromone and heuristic function exponents (ACO-3) notably enhances path planning capability, reducing the optimal path length to 29.799, lowering the average path length to 31.053, and decreasing the number of iterations to convergence to 23.

With the improved heuristic function (ACO-4), the algorithm shows significantly enhanced stability, achieving consistency between average and optimal path lengths (both 29.799), further improving the convergence speed to 18 iterations, and reducing the number of turns to 3. Finally, through the improved pheromone evaporation mechanism (IEACO), the algorithm’s performance reaches optimality, maintaining the optimal path length (29.799) and solution stability while improving the convergence speed to only 9 iterations and achieving the minimum number of turns at 2.

These simulation results clearly demonstrate the performance enhancement contribution of each improvement step. From the initial ACO to the final IEACO, the algorithm achieves significant improvements through its non-uniform initial pheromone distribution, state transition probability optimization, adjustment of the pheromone and heuristic function exponents, improved heuristic function, and pheromone evaporation mechanism optimization, improving the key metrics of path length, convergence speed, and path smoothness to develop a superior path planning algorithm.

### 5.2. Simulation II

In this section, the performance of IEACO is evaluated through comparative simulations with IAACO [38]. The simulations are conducted in two 20×20 spatial environment models, consistent with the configurations reported in the literature [38]. The start point S (denoted by a red pentagram) and target point T (denoted by a blue pentagram) are positioned at indices 1 (top-left corner) and 400 (bottom-right corner) of the environmental model, respectively.

The algorithmic parameters employed in the simulations are summarized in Table 5. To ensure experimental consistency, common parameters are maintained across ACO, IACO, IAACO, and IEACO at identical values (e.g., M = 50, K = 100). Algorithm-specific parameters in IACO and IAACO, such as the angle guidance factor weight coefficient (λ) and adjustment coefficient (k), are set to their optimal values as reported in the literature [38].

**Figure 18:**
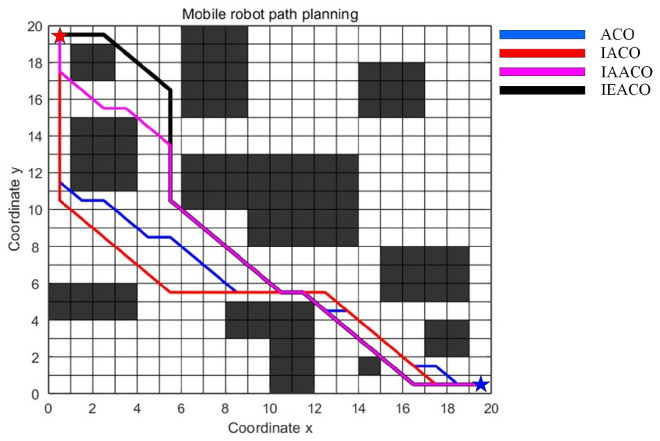
Optimal paths of the four algorithms on the simple map.

**Figure 19:**
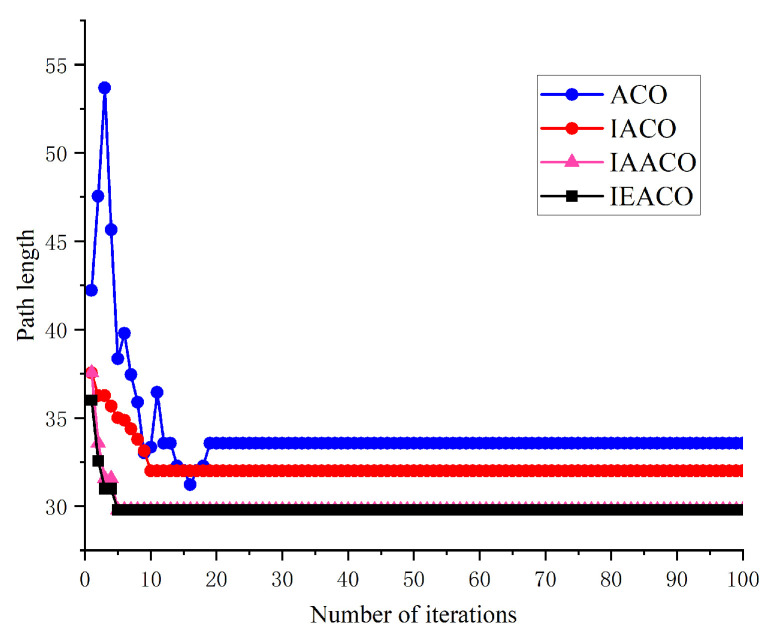
Convergence iteration curves of the four algorithms on the simple map.

**Figure 20:**
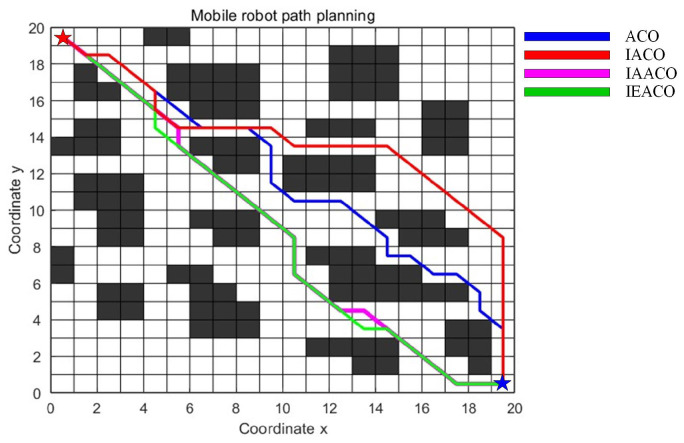
Optimal paths of the four algorithms on the complex map.

**Figure 21:**
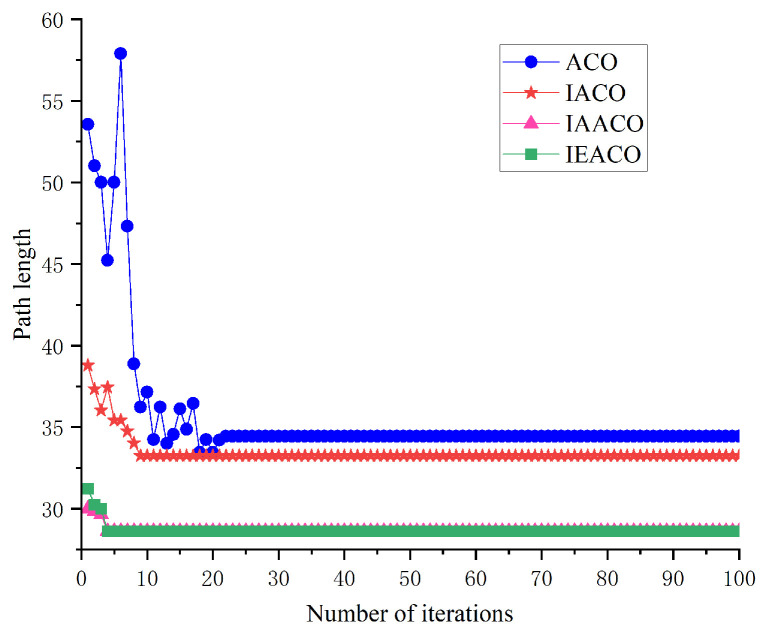
Convergence iteration curves of the four algorithms on the complex map.

The comparative results for both spatial environments are presented in Table 6 and Table 7, with the detailed optimal path planning and optimal path convergence of simple map outcomes shown in Figure 18 and Figure 19. The algorithms’ optimal path planning and optimal path convergence characteristics of complex map are depicted in Figure 20 and Figure 21.

The simulation results demonstrate that the proposed IEACO exhibits significant advantages across all performance metrics. In terms of path length, IEACO achieves the optimal path length (29.78), outperforming traditional ACO (33.56) and surpassing IACO (32.00) and IAACO (29.80). Notably, IEACO demonstrates perfect stability in repeated experiments with a path length standard deviation of 0, which is significantly superior to the other three algorithms. Regarding convergence performance, both IEACO and IAACO exhibit excellent convergence speeds, achieving optimal solutions within 5 iterations, with the average convergence iterations (6.5 and 6.4, respectively) being comparable. This performance significantly surpasses that of IACO (best: 6, average: 9.8) and traditional ACO (best: 14, average: 23.1). It is worth noting that IEACO maintains rapid convergence while ensuring solution stability. In terms of path smoothness, IEACO generates paths with 6 turns; although slightly higher than IACO’s 4, this result is superior to IAACO’s 8 and ACO’s 12. Considering IEACO’s significant advantages in path length and convergence performance, this minor increase in the number of turns represents an acceptable trade-off. A comprehensive comparison indicates that IEACO achieves the shortest path length, fastest convergence speed, and most stable algorithmic performance while maintaining a relatively low number of turns, demonstrating comprehensive advantages in path planning problems. Compared to the other three algorithms, IEACO achieves optimal or near-optimal levels in key indicators such as solution quality, convergence efficiency, and stability, providing a more efficient and reliable solution for path planning problems.

The simulation results in the complex map environment further validate the superior performance of IEACO. Regarding path length, both IEACO and IAACO achieve the optimal path length of 28.63, representing major reductions compared to ACO (31.80) and IACO (32.00). Notably, IEACO demonstrates perfect stability across multiple experiments (standard deviation of 0); despite achieving the same optimal path length, IAACO shows a standard deviation of 0.7497, indicating IEACO’s unique advantage in solution stability. In terms of convergence performance, IEACO and IAACO again demonstrate significant advantages, both achieving optimal solutions within 3 iterations, with average convergence iterations of 4.5 and 4.0, respectively. This performance substantially surpasses that of IACO (average 9.3) and the traditional ACO algorithm (average 27.4), demonstrating that the improved algorithms maintain efficient convergence characteristics even in a more complex environment. Regarding path smoothness, both IEACO and IAACO produce solutions with 7 turns, showing significant improvement over IACO’s 9 and ACO’s 17. This indicates that IEACO can find the shortest path in complex environment while maintaining good path smoothness.

### 5.3. Simulation III

**Figure 22:**
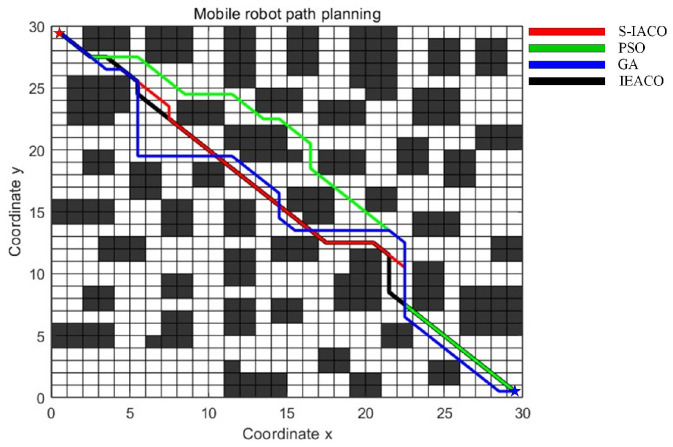
Optimal paths of S-IACO, PSO, GA, and IEACO.

In this section, IEACO’s performance is evaluated on a 30×30 grid map through a comparative analysis with S-IACO [39]. The environmental model from S-IACO [39] is precisely replicated to ensure experimental validity, as illustrated in Figure 22. The start point S (denoted by a red pentagram) and target point T (denoted by a blue pentagram) are positioned at indices 1 (top-left corner) and 900 (bottom-right corner). To establish statistical significance, ten independent simulation experiments were conducted for each algorithm. The algorithmic parameters for both S-IACO and IEACO were configured according to the optimal values reported in [39], as detailed in Table 8.

**Figure 23:**
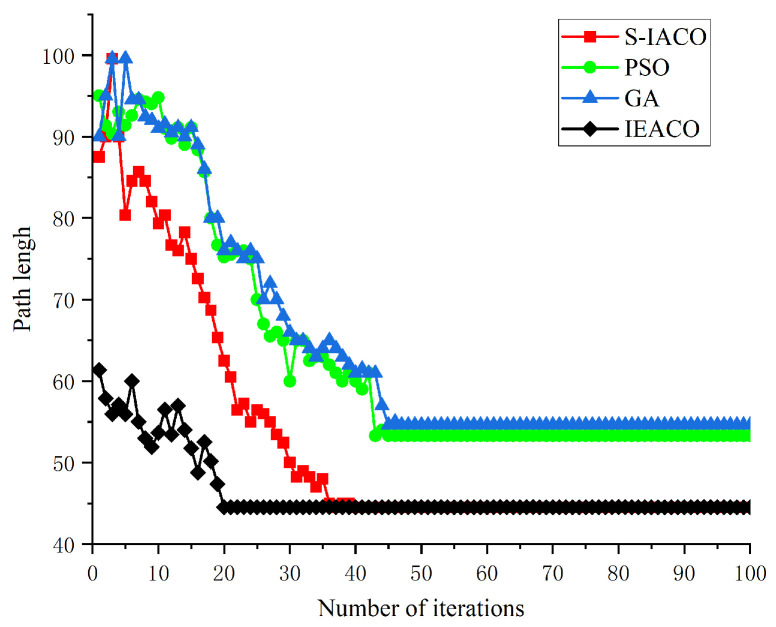
Convergence iteration curves of S-IACO, PSO, GA, and IEACO.

Table 9 shows the results of the four algorithms in ten simulations, including the path length and number of convergence iterations. Figure 22 and Figure 23 present the optimal results of the simulations. These results demonstrate the significant advantages of IEACO in terms of two key metrics, namely, convergence performance and path planning quality. Regarding convergence iterations, IEACO exhibits exceptional stability and efficiency, maintaining consistent convergence within 20 iterations across all ten experiments. In contrast, the other algorithms show notably slower convergence rates; PSO requires an average of 46 iterations (range: 45–48), GA requires an average of 50 iterations (range: 47–53), and S-IACO requires an average of 40 iterations (range: 38–42). This demonstrates IEACO’s significant advantage in convergence speed. In terms of path length, IEACO consistently maintains an optimal path length of 44.52, achieving the same optimization level as S-IACO. This result significantly outperforms PSO (53.29) and GA (54.63). Notably, both IEACO and S-IACO achieve shorter paths while maintaining completely consistent path lengths across all ten simulations, demonstrating exceptional solution stability. Comprehensive analysis indicates that IEACO maintains the optimal path length while achieving faster convergence, demonstrating the significant advantages of our proposed improvements. The simulation results validate IEACO’s comprehensive superiority over traditional intelligent optimization algorithms (PSO and GA) and improved ant colony algorithm (S-IACO) in path planning problems, particularly in terms of stable convergence characteristics and path optimization capability.

### 5.4. Simulation IV

**Figure 24:**
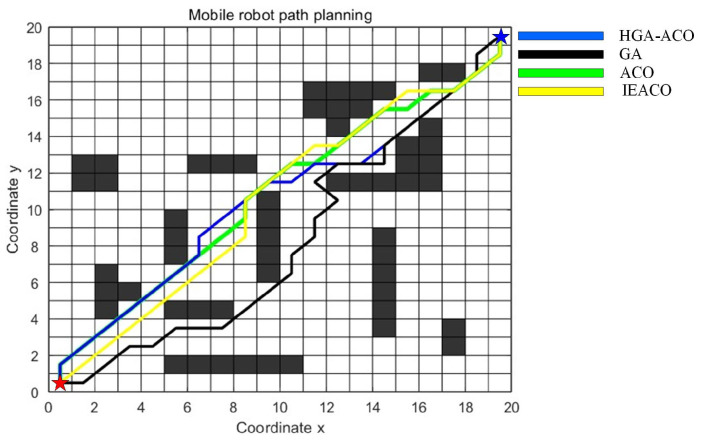
Optimal paths of HGA-ACO, GA, ACO, and IEACO.

**Figure 25:**
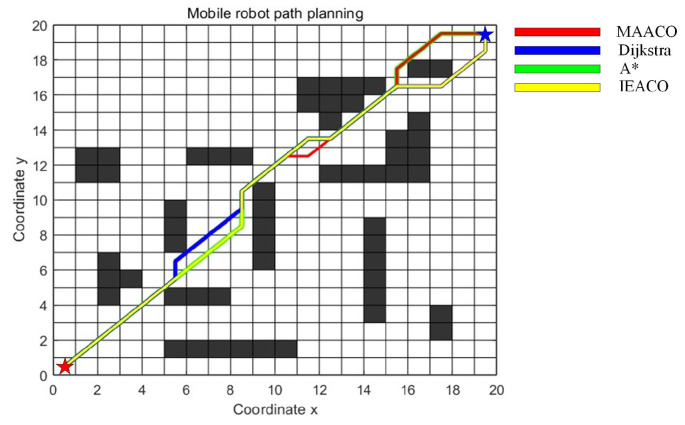
Optimal paths of the MAACO, Dijkstra, A*, and IEACO algorithms.

In this section, IEACO’s performance is evaluated through a comparative analysis with HGA-ACO [40] and MAACO [41]. The simulation utilizes a 20×20 spatial environment model, consistent with the configurations employed in the HGA-ACO [40] and MAACO [41] studies. As illustrated in Figure 24 and Figure 25, the start point S (denoted by a red pentagram) and target point T (denoted by a blue pentagram) are positioned at coordinates (0.5, 0.5) and (19.5, 19.5), corresponding to indices 381 and 20, respectively. To maintain the relative accuracy of the different algorithms’ results, the common parameters of the different algorithms were set to be consistent and the different parameters were set to their respective optimal values. The specific parameter settings are shown in Table 10. Based on the grid map spatial environment model, we independently ran each algorithm 20 times.

The simulation results (Table 11) demonstrate the comprehensive performance advantages of the IEACO algorithm when compared with the other six. Regarding path length, IEACO achieves an optimal path length of 28.63, comparable to HGA-ACO, A*, Dijkstra, and MAACO and superior to GA (30.63) and ACO (29.46). Notably, IEACO, A*, Dijkstra, and MAACO demonstrate perfect path length stability, with standard deviations of zero. In terms of convergence performance, IEACO exhibits optimal convergence characteristics, achieving the optimal solution in just six iterations, with an average convergence of 6.6 and a small standard deviation of 0.1243. This performance significantly surpasses the other iterative algorithms, including GA (36), HGA-ACO (10), ACO (65), and MAACO (best: 7, average: 8.5, standard deviation: 0.2036). Regarding path smoothness, IEACO achieves seven turns, equivalent to A* and MAACO and superior to the others (Dijkstra: 9, ACO: 11, HGA-ACO: 8, GA: 16). This indicates that IEACO maintains good path smoothness while ensuring shortest path optimization. In conclusion, the simulation results demonstrate that IEACO achieves optimal or near-optimal performance across the three key metrics of path length optimality, convergence speed, and path smoothness. In particular, IEACO shows distinct advantages in convergence performance while maintaining high solution stability, validating its comprehensive superiority in path planning problems.

### 5.5. Experiment

**Figure 26:**
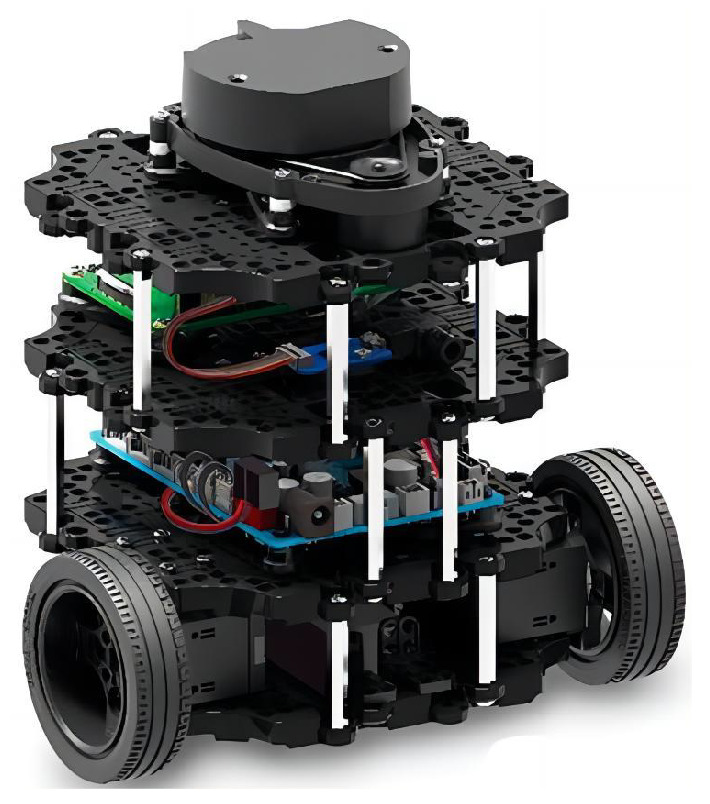
Turtlebot3-Burger.

To validate the effectiveness of IEACO in real-world environments, we designed a physical experiment based on the TurtleBot3-Burger mobile robot platform, as shown in Figure 26. The TurtleBot3-Burger is an open-source platform that is widely used in robotics research, providing an ideal experimental vehicle for this study thanks to its modular design and rich sensor configuration. Integrating the proposed IEACO algorithm into the ROS control system of the TurtleBot3-Burger mobile robot allowed us to directly compare the performance differences between our proposed algorithm and existing methods on actual hardware.

**Figure 27:**
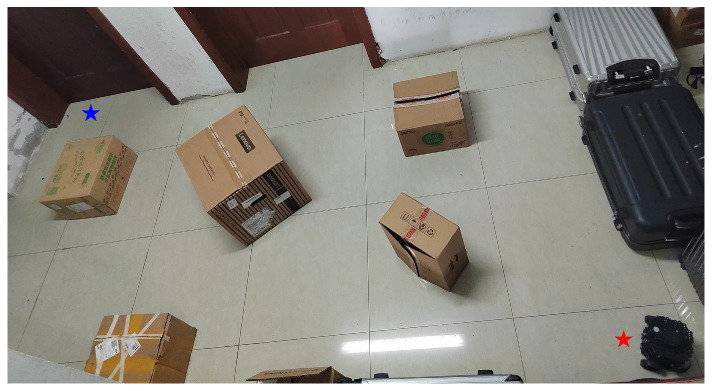
Real-world experimental environment.

The positions of the start point and target point in the experimental setup are shown in Figure 27; the red pentagram represents the start point, while the blue pentagram marks the target point. To comprehensively evaluate the performance of IEACO in engineering applications, we selected the widely used A* and Dijkstra algorithms for this study. Through comparative analysis with these two benchmark algorithms, the advantages of IEACO over widely-used path planning algorithms can be systematically evaluated in terms of key metrics such as path length and number of turns. This comparison also highlights the potential value of IEACO in practical engineering applications.

**Figure 28:**
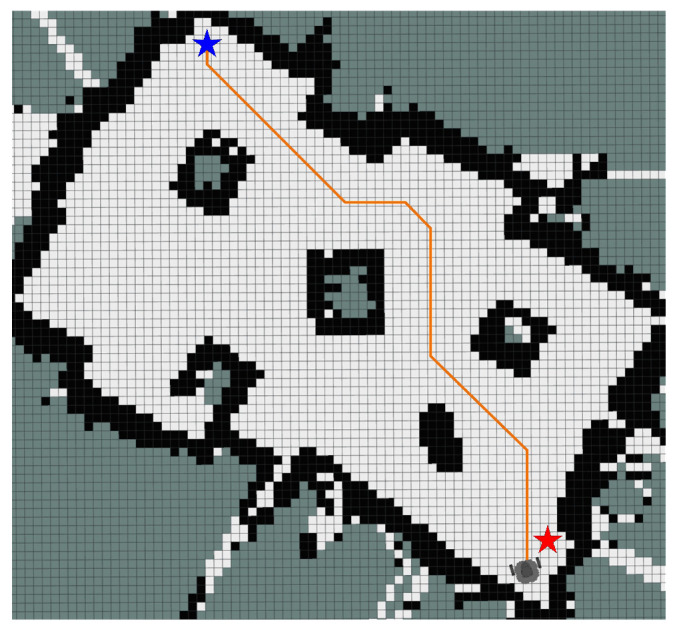
Optimal path of IEACO algorithm.

**Figure 29:**
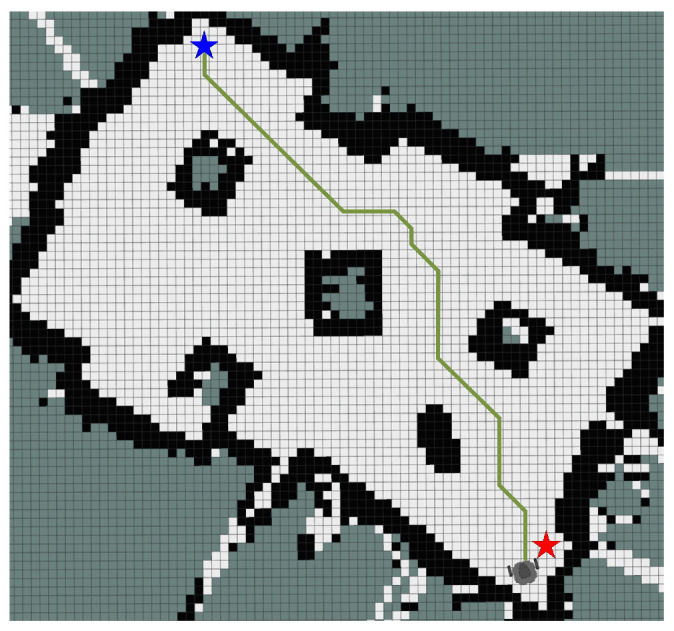
Optimal path of A* algorithm.

**Figure 30:**
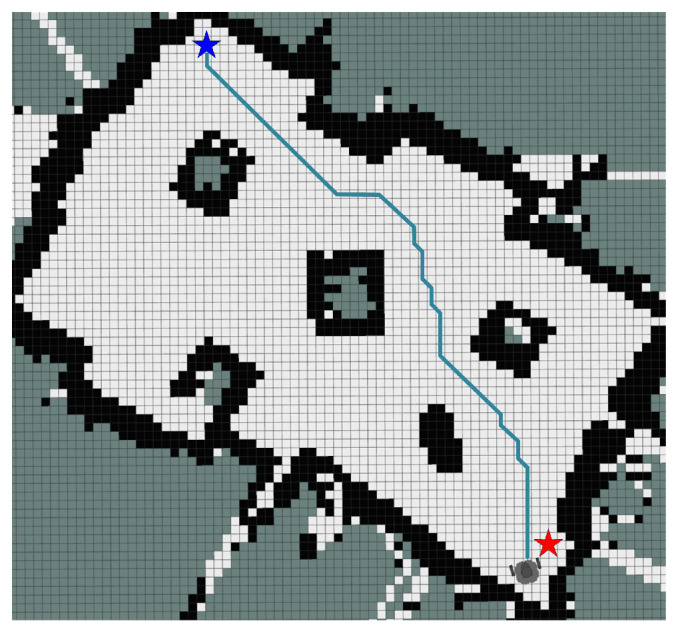
Optimal path of Dijkstra algorithm.

To ensure the statistical significance and reliability of the results, each algorithm was independently executed five times in each experimental scenario. Table 12 summarizes the performance metrics for each algorithm across the five experiments, including key parameters such as path length and number of turns. Additionally, Figure 28, Figure 29 and Figure 30 presents the optimal paths generated by each algorithm, facilitating intuitive comparison. The side length of each grid is 0.05 m.

The experimental results demonstrate that IEACO outperforms the traditional A* and Dijkstra algorithms across all key performance metrics. Regarding path length, IEACO achieves the shortest optimal path length of 3.51m, superior to both A* (3.56 m) and Dijkstra (3.62 m). More significantly, IEACO exhibits perfect stability across multiple experiments, with a path length standard deviation of 0, while the A* and Dijkstra algorithms show respective standard deviations of 0.075 and 0.095 and mean path lengths of 3.65 and 3.73. In terms of path smoothness, IEACO requires only six turns to complete path planning, significantly outperforming A* with 10 and Dijkstra with 16. This indicates that IEACO not only finds shorter paths but also generates smoother motion trajectories. Comprehensive analysis shows that IEACO demonstrates significant advantages in the two key metrics of path length optimality and stability as well as in path smoothness, providing a superior solution for path planning problems. These results conclusively validate the superior performance of IEACO compared to traditional path planning algorithms.

Our comprehensive results from both simulations and real-world experiment demonstrate the superior performance of the proposed IEACO algorithm in path planning tasks. IEACO achieves performance enhancements through optimization of the initial pheromone distribution strategies, improved state transition probability calculations, and enhancements to the heuristic function and exponents. These innovative improvements work together to accelerate algorithm convergence while effectively reducing the path length, number of iterations to convergence, and number of turns. Notably, incorporation of the dynamic pheromone evaporation mechanism effectively mitigates the problem of the algorithm becoming trapped in local optima, enhancing IEACO’s capacity to generate the globally optimal path and significantly improving the solution quality. Our experimental data indicate that IEACO exhibits significant improvements across key performance metrics compared to a variety of existing methods. Its innovative enhancements endow the IEACO algorithm with robust adaptive capabilities and exceptional performance in global path planning tasks, establishing a solid foundation for its deployment in practical engineering applications.

## 6. Conclusions and Future Work

### 6.1. Conclusions

This paper has presented the intelligently enhanced ant colony optimization algorithm (IEACO), which is aimed at improving the performance of traditional and colony optimization in global path planning for mobile robots. The proposed algorithm incorporates six key innovations: (1) optimization of the initial pheromone distribution strategy to enhance early-stage search efficiency; (2) integration of an ε-greedy mechanism to optimize state transition probability, ensuring a dynamic balance between exploration and exploitation; (3) introduction of exponential adaptive adjustment strategies to improve the algorithm’s adaptability across various scenarios; (4) development of a composite heuristic function that combines target distance and turning angles for enhanced path evaluation accuracy; (5) design of a dynamic pheromone update mechanism to effectively prevent premature convergence to local optima; and (6) establishment of a multi-objective evaluation framework to achieve more comprehensive path optimization.

Extensive simulation and experimental evaluations were conducted to validate the advantages of IEACO. First, simulations on the Matlab platform verified the effectiveness of the individual improvements incorporated into IEACO. Subsequently, the algorithm’s computational efficiency and path planning quality were assessed through comparative analyses with different state-of-the-art algorithms on grid maps of different scales. Additionally, practical experimental evaluation was carried out using the ROS platform and a Turtlebot3-Burger mobile robot, demonstrating IEACO’s performance superiority in real-world applications compared to current path planning algorithms commonly used in engineering practice.

The results show that IEACO integrates the above improvements into a coherent framework that outperforms traditional ACO in terms of path length, convergence speed, solution stability, energy consumption, and computational efficiency. The proposed algorithm shows significant potential for real-world applications such as autonomous navigation in complex environments, providing a promising approach for mobile robotics.

### 6.2. Future Work

The simulation and experimental results in this paper demonstrate that IEACO significantly outperforms existing algorithms in key performance metrics, including path length, convergence speed, and planning stability. However, there is still room for further optimization and extension of this research. First, while the proposed algorithm has shown promising results, its performance could be further validated in more challenging environments by increasing the complexity of the experimental scenarios or deploying it on actual mobile robot platforms in industrial or real-world settings. Second, integrating IEACO with curve optimization methods such as cubic B-spline curves could help to transform discrete paths into continuous trajectories that better align with the kinematic characteristics of mobile robots. Third, combining IEACO with local path planning methods is an important avenue for future research. This would improve the algorithm’s adaptability in dynamic and uncertain environments, helping to ensure real-time responsiveness and efficiency in various application contexts. Future work will also focus on addressing quantitative analysis of energy consumption and time complexity, where we plan to develop a robotic experimental platform integrated with multi-dimensional sensors to acquire precise metrics that will enable more rigorous evaluation and validation.

## Figures and Tables

**Table 1:** Description of parameters.

Parameter	Description
M	Number of ants
α	Pheromone exponent
β	Heuristic function exponent
ρ	Pheromone evaporation factor
Q	Pheromone strength value
K	Maximum number of iterations
$L_{m}$	Path length for ant m
$\eta_{ij}\left(k\right)$	Heuristic function at time k
$\tau_{ij}\left(k\right)$	Pheromone concentration between the i-th point and j-th point at time k
$\Delta \tau_{ij}^{m}\left(k\right)$	Pheromone increment between point i and point j at time k for ant m
$p_{ij}^{m}\left(k\right)$	Probability that ant m moves from the i-th point to the j-th point in the k-th iteration
$S\in allowed_{m}$	Potentially accessible grid cells of ant m around the next point

**Table 2:** Improvement methods for the different ACO variants.

Improving the Methods of ACO	Variants
Traditional ACO	ACO
ACO with the improvement method proposed in Section 4.1	ACO-1
(Non-uniform distribution of initial pheromone)	
ACO with the improvement methods proposed in Section 4.1 and Section 4.2	ACO-2
(Non-uniform distribution of initial pheromone)	
(State transition probability improved by the ε-greedy strategy)	
ACO with the improvement methods proposed in Section 4.1, Section 4.2 and Section 4.3	ACO-3
(Non-uniform distribution of initial pheromone)	
(State transition probability improved by the ε-greedy strategy)	
(Optimization of the exponents α and β)	
ACO with the improvement methods proposed in Section 4.1, Section 4.2, Section 4.3 and Section 4.4	ACO-4
(Non-uniform distribution of initial pheromone)	
(State transition probability improved by the ε-greedy strategy)	
(Optimization of the exponents α and β)	
(Multi-objective heuristic function)	
ACO with the improvement methods proposed in Section 4.1, Section 4.2, Section 4.3 and Section 4.4 and Section 4.5	IEACO
(Non-uniform distribution of initial pheromone)	
(State transition probability improved by the ε-greedy strategy)	
(Optimization of the exponents α and β)	
(Multi-objective heuristic function)	
(Adjusting the global pheromone update mechanism)	

**Table 3:** Parameter settings of the traditional ACO algorithm and variants.

Algorithm	M	K	α	β	Q	ρ
ACO	50	100	1	7	1	1
Variants	50	100	$\alpha^{\prime }$	$\beta^{\prime }$	$Q^{\prime }$	$\rho^{\prime }$

**Table 4:** Simulation results of the ACO algorithm and variants.

Algorithm	Optimal Path Length	Average Path Length	Number of Convergence Iterations	Number of Turns
ACO	33.556	34.675	-	11
ACO-1	31.798	33.986	72	8
ACO-2	31.213	33.142	31	5
ACO-3	29.799	31.053	23	6
ACO-4	29.799	29.799	18	3
IEACO	29.799	29.799	9	2

**Table 5:** Parameter settings for ACO, IACO, IAACO, and IEACO.

Algorithm	M	K	λ	k	α	β	$\delta_{1}$	$\delta_{2}$	$R_{s}$	$\delta_{0}$	Q	$K_{L}$	$K_{S}$	$K_{E}$	ρ
ACO	50	100	7	0.9	1	7	0.1	0.9	0.5	0.15	2.5	0.7	0.1	0.2	0.2
IACO	50	100	7	0.9	1	7	0.1	0.9	0.5	0.15	2.5	0.7	0.1	0.2	-
IAACO	50	100	7	0.9	1	7	0.1	0.9	0.5	0.15	2.5	0.7	0.1	0.2	-
IEACO	50	100	-	-	$\alpha^{\prime }$	$\beta^{\prime }$	-	-	-	-	$Q^{\prime }$	-	-	-	$\rho^{\prime }$

**Table 6:** Statistical results of ACO, IACO, IAACO, and IEACO on the simple map.

Algorithm	Path Length			Number of Convergence Iterations			Number of Turns
	Optimal Path	Mean	Std.	Best	Mean	Std.	
ACO	33.56	36.609	3.4189	14	23.1	6.789	12
IACO	32.00	33.22	0.9938	6	9.8	3.3407	4
IAACO	29.80	30.122	0.3953	5	6.4	1.0168	8
IEACO	29.78	29.78	0	5	6.5	1.0243	6

**Table 7:** Statistical results of ACO, IACO, IAACO, and IEACO on the complex map.

Algorithm	Path Length			Number of Convergence Iterations			Number of Turns
	Optimal Path	Mean	Std.	Best	Mean	Std.	
ACO	31.80	33.764	2.9262	17	27.4	5.5353	17
IACO	32.00	33.26	0.8294	6	9.3	3.6069	9
IAACO	28.63	29.505	0.7497	3	4	0.8944	7
IEACO	28.63	28.63	0	3	4.5	1.3512	7

**Table 8:** Parameter settings for S-IACO and IEACO.

Algorithm	M	K	α	β	Q	ρ	ξ
S-IACO	50	100	1	7	2	2	0.5
IEACO	50	100	$\alpha^{\prime }$	$\beta^{\prime }$	$Q^{\prime }$	$\rho^{\prime }$	-

**Table 9:** Statistical results of PSO, GA, S-IACO, and IEACO.

	Number of Convergence Iterations				Path Length			
**Algorithm**	**PSO**	**GA**	**S-IACO**	**IEACO**	**PSO**	**GA**	**S-IACO**	**IEACO**
1	45	51	39	20	53.29	54.63	44.52	44.52
2	47	50	39	20	53.29	54.63	44.52	44.52
3	47	51	40	20	53.29	54.63	44.52	44.52
4	46	49	38	20	53.29	54.63	44.52	44.52
5	45	48	42	20	53.29	54.63	44.52	44.52
6	47	53	40	20	53.29	54.63	44.52	44.52
7	48	53	41	20	53.29	54.63	44.52	44.52
8	45	47	38	20	53.29	54.63	44.52	44.52
9	45	50	42	20	53.29	54.63	44.52	44.52
10	46	51	40	20	53.29	54.63	44.52	44.52
Average	46	50	40	20	53.29	54.63	44.52	44.52

**Table 10:** Parameter settings for ACO, HGA-ACO, MAACO, and IEACO.

Algorithm	M	K	α	β	Q	ρ	$P_{c}$	$P_{m}$	a	b	A	B	$N_{c}$	$w_{\mathrm{hmax}}$	$w_{\mathrm{hmin}}$	$q_{0-\mathrm{initial}}$
ACO	50	100	1	7	1	0.3	-	-	-	-	-	-	-	-	-	-
HGA-ACO	50	100	1	7	1	0.3	0.8	0.2	1	7	0	100	100	-	-	-
MAACO	50	100	1	7	1	0.3	-	-	1	-	-	-	-	0.9	0.2	0.5
IEACO	50	100	$\alpha^{\prime }$	$\beta^{\prime }$	$Q^{\prime }$	$\rho^{\prime }$	-	-	-	-	-	-	-	-	-	-

**Table 11:** Statistical data of the seven algorithms.

Algorithm	Path Length			Number of Convergence Iterations			Number of Turns
	Optimal Path	Mean	Std.	Best	Mean	Std.	
GA	30.63	-	-	36	-	-	16
HGA-ACO	28.63	-	-	10	-	-	8
ACO	29.46	29.46	0	65	-	-	11
A*	28.63	28.63	0	-	-	-	7
Dijkstra	28.63	28.63	0	-	-	-	9
MAACO	28.63	28.63	0	7	8.5	0.2036	7
IEACO	28.63	28.63	0	6	6.6	0.1243	7

**Table 12:** Experimental results of the IEACO, A*, and Dijkstra algorithms.

Algorithm	Path Length			Number of Turns
	Optimal Path	Mean	Std	
IEACO	3.51	3.51	0	6
A*	3.56	3.65	0.075	10
Dijkstra	3.62	3.73	0.095	16

## Data Availability

All data are contained within the article.
